# DAXX adds a *de novo* H3.3K9me3 deposition pathway to the histone chaperone network

**DOI:** 10.1016/j.molcel.2023.02.009

**Published:** 2023-04-06

**Authors:** Massimo Carraro, Ivo A. Hendriks, Colin M. Hammond, Victor Solis-Mezarino, Moritz Völker-Albert, Jonas D. Elsborg, Melanie B. Weisser, Christos Spanos, Guillermo Montoya, Juri Rappsilber, Axel Imhof, Michael L. Nielsen, Anja Groth

**Affiliations:** 1 Novo Nordisk Foundation Center for Protein Research (CPR), Faculty of Health and Medical Sciences, University of Copenhagen, Copenhagen, Denmark; 2 Biotech Research and Innovation Centre (BRIC), Faculty of Health and Medical Sciences, University of Copenhagen, Copenhagen, Denmark; 3 EpiQMAx GmbH, Planegg, Germany; 4 Wellcome Centre for Cell Biology, University of Edinburgh, Edinburgh, UK; 5 Technische Universität Berlin, Chair of Bioanalytics, Berlin, Germany; 6 Faculty of Medicine, Biomedical Center, Protein Analysis Unit, Ludwig-Maximilians-Universität München, Planegg-Martinsried, Germany

**Keywords:** ASF1, DAXX, NASP, HJURP, histone chaperone, protein network, nucleosome assembly, heterochromatin, gene silencing, proteomics, epigenetic

## Abstract

A multitude of histone chaperones are required to support histones from their biosynthesis until DNA deposition. They cooperate through the formation of histone co-chaperone complexes, but the crosstalk between nucleosome assembly pathways remains enigmatic. Using exploratory interactomics, we define the interplay between human histone H3–H4 chaperones in the histone chaperone network. We identify previously uncharacterized histone-dependent complexes and predict the structure of the ASF1 and SPT2 co-chaperone complex, expanding the role of ASF1 in histone dynamics. We show that DAXX provides a unique functionality to the histone chaperone network, recruiting histone methyltransferases to promote H3K9me3 catalysis on new histone H3.3–H4 prior to deposition onto DNA. Hereby, DAXX provides a molecular mechanism for *de novo* H3K9me3 deposition and heterochromatin assembly. Collectively, our findings provide a framework for understanding how cells orchestrate histone supply and employ targeted deposition of modified histones to underpin specialized chromatin states.

## Highlights

• Panoramic view of the histone H3–H4 chaperone interaction network
• Characterization of histone co-chaperone complexes and histone variant specificities
• DAXX recruits new H3.3–H4 into complexes with H3K9me3 writers and readers
• DAXX stimulates H3.3K9me3 catalysis during the supply of new histones to chromatin

## Graphical abstract

**Figure:**
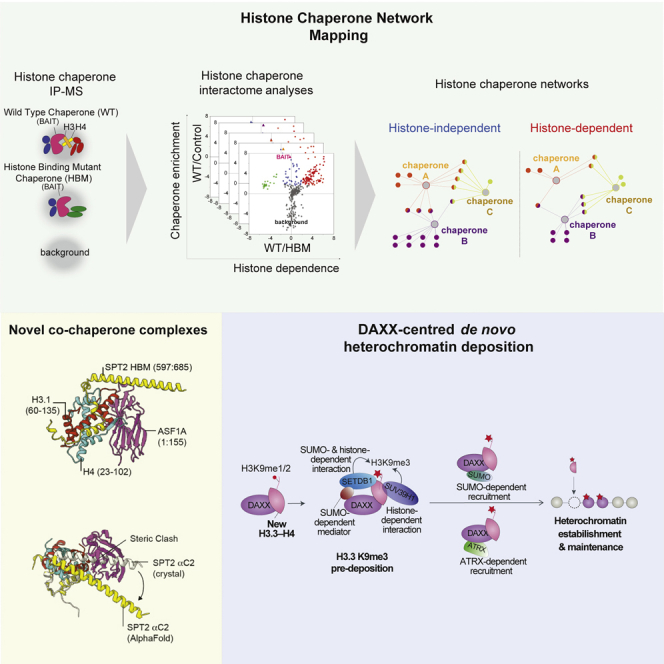

## Teaser

Carraro et al. charted the histone H3–H4 chaperone network, revealing co-chaperone relationships involved in nucleosome assembly and unveiling that histone chaperone DAXX promotes lysine 9 tri-methylation of new histone H3.3 prior to deposition onto DNA. With its functionality, DAXX provides a molecular mechanism for *de novo* heterochromatin assembly.

## Introduction

In eukaryotic cells, genomic DNA is packaged with histone proteins into chromatin which regulates genome function and stability. The basic unit of chromatin is the nucleosome, formed by 147 base pairs of DNA wrapped around an octameric complex of histones H3, H4, H2A, and H2B.^1^ Nucleosomes are modified through histone post-translational modifications (PTMs) and the substitution of core histones with histone variants. This histone-based epigenetic information drives chromatin functionality, regulating gene expression, silencing repetitive elements, and instructing DNA damage response pathways.^2^^,^^3^ To allow the passage of the molecular machines that transcribe, replicate, or repair the DNA template, nucleosomes are disassembled and reassembled, and this is complemented with new histone deposition pathways that maintain nucleosome density.^4^^,^^5^^,^^6^ This is especially important during DNA replication where deposition of new histones is required to maintain nucleosome density on daughter DNA strands.^7^

Histone supply and chromatin dynamics are supported by a structurally diverse set of proteins called histone chaperones.^4^^,^^5^^,^^6^ Histone chaperones shield the interactions of histones with DNA/RNA in a manner that only proper nucleosome contacts can out-compete,^8^ thereby promoting the ordered assembly and disassembly of nucleosomes. Histone chaperones often collaborate, forming histone-dependent co-chaperone complexes.^4^ In these complexes, multiple chaperones simultaneously associate with the same histone-fold dimer or tetramer providing a more complete shield around the histone substrate,^9^^,^^10^^,^^11^^,^^12^ and potentially promoting nucleosome assembly.^9^ Histone chaperones can also combine through direct histone-independent interactions to provide multivalency to their chaperoning functionality or to mediate histone handover events. Histone chaperone functionality is also integrated within chromatin remodelers, histone-modifying enzymes, heat shock molecular chaperones, and DNA/RNA polymerases and helicases.^4^^,^^13^^,^^14^ Thus histone chaperone functionality has a broad influence on the chromatin state of a cell.

In mammals, the incorporation of the canonical histone H3 (H3.1 and H3.2) and its replacement variants (H3.3 and CENPA) have profound effects on chromatin organization,^15^ and each variant associates with partially distinct chaperone systems. After translation, newly synthesized canonical histones H3.1/2 and variant H3.3 are co-folded with histone H4 by the histone chaperone DNAJC9.^13^ Folded H3–H4 dimers are then handled by histone chaperone NASP, which protects a soluble pool of histones from chaperone-mediated autophagy.^16^^,^^17^^,^^18^ The somatic isoform of NASP (sNASP) also functions in the HAT-1 complex with histone chaperones RbAp46 (RBBP7) and the histone acetyltransferase HAT1.^19^ The HAT-1 complex promotes histone H4 K5/K12 acetylation of H3–H4 dimers that associate with ASF1a/b and are imported into the nucleus via Importin-4/IPO4.^4^^,^^5^ ASF1a/b coordinates *de novo* H3–H4 supply to the CAF-1 and HIRA complexes,^4^^,^^5^ which are responsible for replication- and transcription-coupled deposition of H3.1/2–H4 and H3.3–H4, respectively.^20^

During histone supply, ASF1 also forms co-chaperone interactions with both MCM2 and TONSL.^9^^,^^11^^,^^21^^,^^22^ MCM2 is part of the CMG helicase complex (CDC4, MCM2-7, and GINS) and functions as a histone recycling factor during DNA replication,^23^^,^^24^ while stabilizing soluble H3–H4 bound by ASF1.^9^ TONSL binds newly synthesized histones through recognition of histone H4 unmethylated at K20,^11^ a mark of post-replicative chromatin that promotes DNA damage repair via homologous recombination.^11^^,^^25^ While ASF1, MCM2, and TONSL are co-chaperone partners,^9^^,^^11^ it is not entirely clear whether TONSL contributes to both H3.1/2 and H3.3–H4 supply pathways in mammals.

At sites of constitutive heterochromatin H3.3–H4 dimers are deposited by the histone chaperone DAXX.^26^^,^^27^^,^^28^ DAXX-mediated H3.3–H4 deposition is essential for maintaining silencing of repetitive DNA elements including telomeres, viral genomes, retrotransposons, imprinted regions,^29^^,^^30^^,^^31^^,^^32^ and also plays a role in preventing replication stress.^33^ Despite the importance of this deposition pathway, the stage of histone supply when DAXX associates with histones remains elusive.^4^ DAXX interacts with the chromatin remodeler ATRX,^34^ the histone methyltransferases SUV39H1^30^ and SETDB1/SETB1/ESET, and the SETDB1-linked co-repressor protein TRIM28/TIF1B/KAP1^29^^,^^35^ during the establishment of heterochromatic silencing. Additionally, DAXX-mediated transcriptional silencing requires DAXX localization to promyelocytic leukemia (PML) nuclear bodies in a SUMOylation dependent manner.^36^ DAXX deposition of H3.3–H4 is required for maintenance of H3K9me3,^29^^,^^30^^,^^37^^,^^38^ a PTM linked to transcriptional silencing.^39^ Why H3.3 deposition is required for H3K9me3 enrichment and heterochromatin silencing remains unclear, especially since H3.3–H4 is also deposited at sites of active transcription by HIRA.^27^ CENPA-H4 dimers are also handled in a variant-specific manner by the histone chaperone HJURP,^40^^,^^41^ which promotes their deposition at centromeric chromatin through the MIS18 complex.^42^^,^^43^

The cooperative nature of histone chaperones in histone metabolism suggests the existence of an interconnected histone chaperone network.^4^ However, the organization of the network and the crosstalk between H3 variant supply pathways has not been systematically studied. Here, we define the topology of the histone chaperone network surrounding key nodes in the histone H3 variant supply chains, providing a rich resource to broaden our understanding of new and existing players in histone chaperone biology. We delineate the crosstalk between different H3–H4 chaperones systems revealing that DAXX operates as a largely independent arm of the histone chaperone network. Through interrogation of DAXX functionality, we demonstrate a route for the delivery of newly synthesized histone H3.3–H4 dimers modified with H3K9me3 to chromatin, unveiling a molecular mechanism for *de novo* heterochromatin assembly.

## Results

### Charting the histone chaperone network

**Figure 1:**
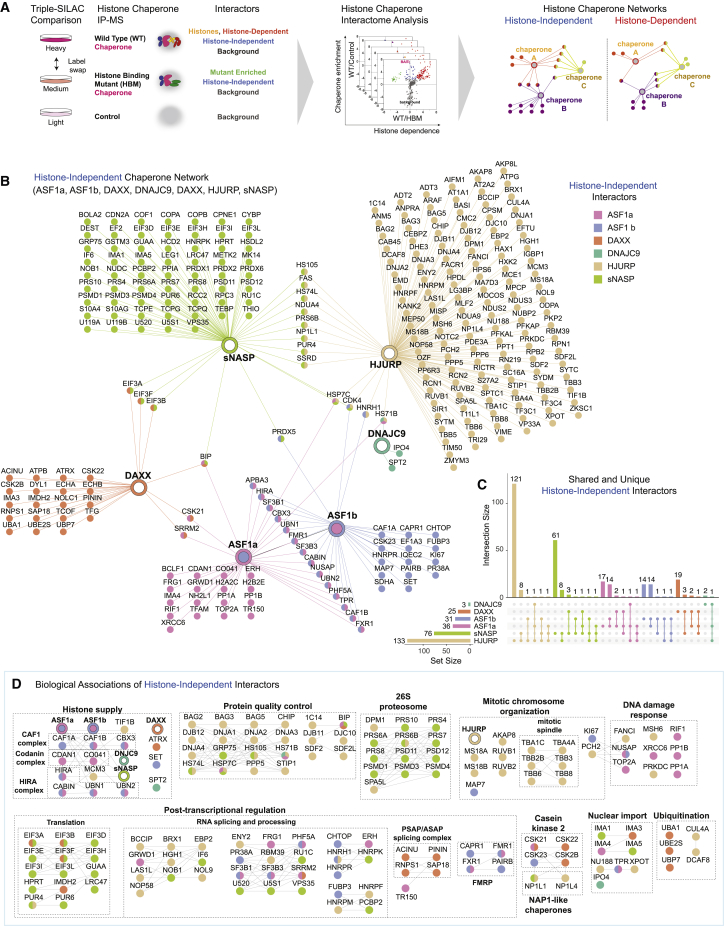
Histone chaperones directly interact with diverse cellular processes (A) The triple SILAC IP-MS strategy for mapping the histone chaperone network. (B) Network analysis of histone-independent interactors identified for ASF1a, ASF1b, NASP, DAXX, HJURP, and DNAJC9. Nodes and edges are colored based on their identification in the different pull-downs. (C) Upset plot showing the overlap of histone-independent interactomes, colored as in (B). (D) Clustering of histone-independent interactors using functional associations annotated in the STRING database and the MCL algorithm. Protein-protein functional associations are shown with a black line according to the string database, protein nodes colored as in (B). See also Figure S2A. (B–D) Proteins are referred to by human UniProt protein identification code. Data generated from n = 4 biological replicates. See also Table S1 and Figures S1 and S2.

To understand the connectivity within the histone chaperone network and identify new co-chaperone relationships, we profiled ASF1a/b, sNASP, HJURP, and DAXX histone-dependent and -independent interactomes. Together this panel of histone chaperones allowed us to monitor the pathways of replication-dependent and -independent nucleosome assembly, soluble histone homeostasis, centromere assembly, and heterochromatin maintenance.^4^^,^^5^ To this end, we compared the interactomes of conditionally expressed wild-type (WT) histone chaperones with their corresponding histone-binding mutant (HBM), and a negative control in triple SILAC IP-MS experiments (stable isotope labeling with amino acids in cell culture, immunoprecipitation coupled to mass spectrometry) (Figure 1A). Based on previous studies, we identified point mutations that disrupt histone binding and profiled the histone dependence of histone chaperone interactomes using proteomic assays (Figure S1A–S1F).^44^^,^^45^^,^^46^^,^^47^ We recently identified DNAJC9 as a new player in histone supply using a similar experimental strategy.^13^

We compared our datasets with findings from the literature, confirming the robust nature of our experimental approach (Figures S1B–S1F, Table S1). For instance, we were able to confirm ASF1a/b histone-dependent interactions with MCM2,^9^^,^^21^ TONSL,^11^ NASP, and the HAT-1 complex (HAT1 and RBBP7), as well as IPO4^,^ (Figures S1B and S1C).^9^^,^^22^ sNASP interactomes corroborated the histone-dependent association with ASF1a/b and HAT-1 complex members^19^^,^^22^^,^^48^ (Figure S1D). Furthermore, ASF1a/b also co-purified CAF1B (CAF-1 p60) and the HIRA complex (HIRA, UBN1/2, and CABIN) independent of histone binding (Figures S1B and S1C), in line previous work.^49^ Finally, our pull-downs confirmed the direct histone-independent interaction of DAXX and ATRX (Figure S1F).^28^^,^^35^

We next performed a network analysis of histone-independent interactors identified in our histone chaperone interactomes (Figure 1B, Table S1), including the published triple SILAC IP-MS datasets for DNAJC9.^13^ Strikingly, most of the histone chaperones analyzed had a largely unique histone-independent interactome, with only a few proteins interacting with multiple chaperones (Figure 1C). To explore the biological themes encoded in these interactomes, we generated a clustered interaction network that integrates the functional associations between interactors from the STRING database and mapped these clusters to known protein complexes and pathways (Figures 1D and S2A). This revealed an enrichment of proteins involved in post-transcriptional regulation and RNA processing in several of the interactomes. For instance, DAXX associates with PSAP and ASAP splicing complexes (SAP18, PININ, RNPS1, and ACINU),^50^ NASP associates with several pre-mRNA-binding proteins (RUC1, U520, U5S1, and HNRPK), and ASF1a/b link to Fragile X syndrome RNA binding proteins (FXR1, FMR1, CAPR1, and PAIRB).^51^ This underscores the integration of histone chaperones with post-transcriptional regulation as an emerging theme in histone chaperone biology.^52^^,^^53^ The enrichment of heat shock molecular chaperones was another common theme shared across datasets, strengthening the functional link between these complementary chaperone systems.^13^ Meanwhile, nuclear import proteins also featured prominently in this analysis. sNASP interacted with the importin proteins IMA1/KPNA2 and IMA5/KPNA1, the latter was recently implicated in the nuclear import of monomeric histones,^54^ which can also be bound in the nucleus by sNASP.^55^ DNAJC9 formed histone-independent interactions with IPO4,^13^ while ASF1 and DAXX associated with the importins IMP4/KPNA3 and IMA3/KPNA4, respectively, potentially providing alternative pathways for the nuclear import of histone H3–H4 dimers.

In contrast, several unique biological processes were enriched by an individual histone chaperone or a select subset of those profiled. Several subunits of the 26S proteasome^56^ were exclusively enriched with sNASP, placing NASP in proximity of both the proteasomal and autophagy-mediated^16^ degradation machineries. Proteins involved in mitotic chromosome segregation (MIS18A, MIS18B, TRIP13, RUVB1, RUVB2, and AKAP8)^57^^,^^58^^,^^59^^,^^60^ and the mitotic spindle^61^ associate with HJURP. These interactions are in line with the histone-binding domain of HJURP being dispensable for its recruitment to the MIS18 complex (MIS18A and MIS18B) and the centromere.^42^^,^^43^^,^^62^ Finally, factors involved in DNA repair pathways were purified with either HJURP (MSH6, FANCI, and PRKDC) or ASF1a (XRCC6, TOP2A, RIF1, and PP1A/B), consistent with the involvement of these histone chaperones in DNA repair pathways.^63^^,^^64^ To cross-validate the isoform specificity of ASF1 interactors, we directly compared ASF1a and ASF1b interactomes (Figure S2B). Corroborating previous findings, ASF1b displayed a preference for the CAF-1 complex^65^ and ASF1a specifically associates with RIF1 in a histone-independent manner,^66^ which suggests a role for RIF1 in histone deposition. Meanwhile, the HIRA complex associates with both ASF1 isoforms, consistent with our network analysis (Figure 1B) and a recent study.^67^

### Histone chaperone cooperation is built through histone-dependent interactions

**Figure 2:**
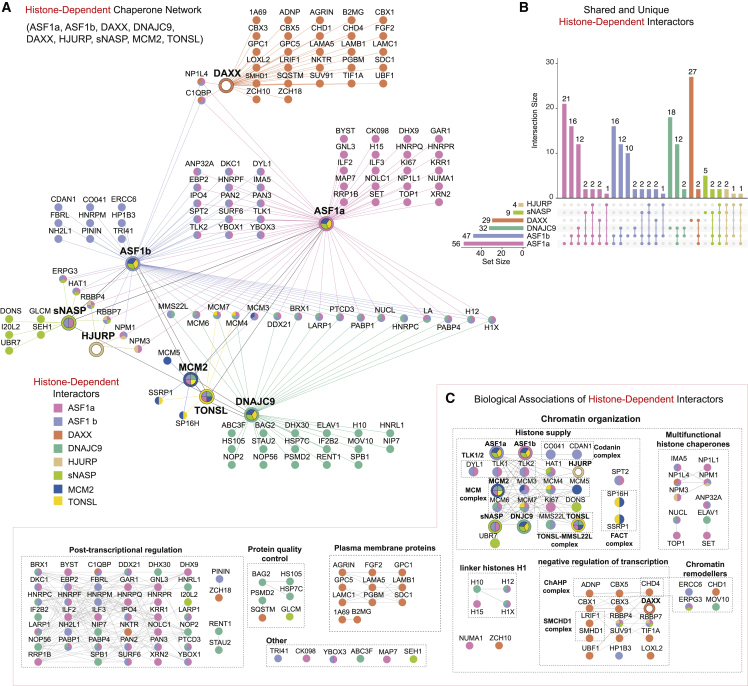
Histone chaperones collaborate through their histone-dependent associations (A) Network analysis of the histone-dependent interactors identified for ASF1a, ASF1b, NASP, DAXX, HJURP, DNAJC9, MCM2, and TONSL. Nodes and edges are colored based on their identifications in the different pull-downs. (B) Upset plot showing the overlaps between histone-dependent interactomes. Protein nodes, edges are colored based on their identifications in the different pull-downs, colored as in (A). (C) Clustering of histone-independent interactors using functional associations annotated in the STRING database and the MCL algorithm. Protein-protein functional associations are shown with a black line according to the string database, protein nodes colored as in (A). (A–C) Proteins are referred to by human UniProt protein identification code. Data generated from n = 4 biological replicates. See also Table S1 and Figure S1.

Next, we compared the histone-dependent interactors identified in our chaperone pull-downs, incorporating published datasets for DNAJC9, MCM2, and TONSL^13^ (Figures 2A and 2B). This analysis revealed a substantial overlap between interactors (Figure 2B). ASF1a and ASF1b shared a large proportion of histone-dependent interactors (34/56 and 34/47, respectively), in line with the similar roles of the two isoforms in nucleosome assembly pathways.^4^^,^^68^ Similarly, DNAJC9 shared several histone-dependent interactors with ASF1a and ASF1b (12/32 and 14/32, respectively), supporting the idea that DNAJC9 operates in parallel to ASF1 in histone supply.^13^ Despite the high degree of overlap between chaperones in the histone-dependent network, we only observed two shared interactions linking DAXX to the rest of the network. These were the histone chaperones NP1L4/NAP1L4 and C1QBP^69^^,^^70^ that also form histone-dependent interactions with ASF1a/b, representing possible links between DAXX and ASF1 nucleosome assembly pathways.

We grouped the histone-dependent proteins into known pathways and complexes highlighting biological processes in histone supply chains (Figure 2C). Again, factors involved in post-transcriptional regulation were enriched, further linking histone chaperone functionality to the RNA processing machinery. DNAJC9, sNASP, and DAXX were associated in a histone-dependent manner with factors involved in protein quality control. DNAJC9 was the only chaperone to contact heat shock molecular chaperones in a histone-dependent manner (HSP74, HSP7C, HS105, and BAG2), supporting its unique role in recruiting these molecular chaperones to soluble histones.^13^ sNASP and DAXX purified with factors involved in autophagy-mediated protein degradation (GLCM/GBA1 and SQSTM, respectively). sNASP is known to protect soluble histones H3 and H4 from chaperone-mediated autophagy.^16^^,^^17^ Therefore, the histone-dependent association of sNASP with the lysosomal protein GLCM,^71^ combined with its histone-independent interactions with the proteasome (Figure 1B), suggests that sNASP may coordinate a protein folding versus degradation decision point for soluble histones.

Meanwhile, DAXX formed histone-dependent interactions with a set of enigmatic proteins, including the proteoglycans (GPC1 and GPC5),^72^ major histocompatibility complex I members (1A69/HLAA and beta-2-microglobulin/B2MG),^73^ and the cargo receptor for selective autophagy SQSTM/p62.^74^ These interactors potentially reflect a role for DAXX in the membrane localization or extracellular secretion of histones.^75^^,^^76^ Otherwise, DAXX–H3–H4 soluble complexes were predominantly associated with factors involved in negative regulation of transcription (CBX5/HP1α, CBX1/HP1β, CBX3/HP1γ, TIF1A/TRIM24, ADNP, SUV91/SUV39H1, SMHD1/SMCHD1, LRIF1, and LOXL2).^77^^,^^78^^,^^79^^,^^80^^,^^81^^,^^82^ As these interactomes were generated from soluble cell fractions, this argues that DAXX specifically recruits factors involved in heterochromatin organization to its H3.3–H4 cargo prior to chromatin deposition.

### Characterization of histone-dependent interactions/histone co-chaperone complexes

**Figure 3:**
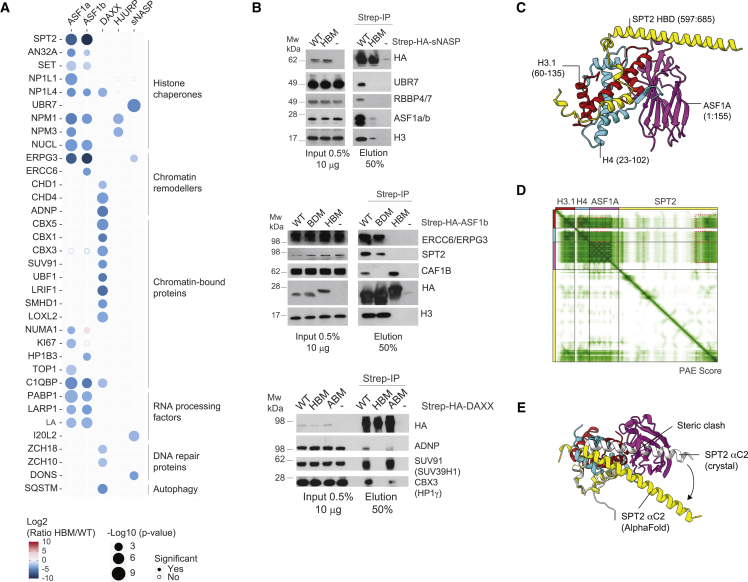
Validation of previously uncharacterized histone-dependent interactors (A) Bubble plot showing histone-dependent interactors across triple SILAC IP-MS interactomes. Proteins are referred to by human UniProt protein identification code. Data generated from n = 4 biological replicates. Colors represent Log2 SILAC ratios (HBM/WT), and radii represent p values. See also Table S1. (B) Pull-downs of Strep-HA-tagged histone chaperones WT, HBM, B domain mutant (BDM), or ATRX-binding mutant (ABM) compared with control purifications (−) from soluble cell extracts probed by western blot. Representative of n = 2 biological replicates. (C) “Local” AlphaFold prediction of SPT2 (yellow) and ASF1A (magenta) histone-binding domains bound to H3.1–H4 (red and cyan) depicting the high-confidence regions of the full-length AlphaFold prediction. (D) Predicted alignment error (PAE) plot showing confidence of residue contacts in the full-length SPT2–H3.1–H4–ASF1A “global” AlphaFold prediction. Red dashed lines indicate high-confidence interactions between protein chains in the “local” prediction shown (left). See also Figures 3A–3C. (E) Alignment of local AlphaFold prediction of SPT2–H3.1–H4–ASF1A (colored as C) to the crystal structure of SPT2–(H3.2–H4)_2_ (white, PDB: 5BS7, with H3.2–H4 omitted for clarity). See also Figures S3D and S3E.

Our proteomic analysis identified several uncharacterized histone co-chaperone complexes (Figure 3A). Histone chaperones co-purified with ASF1a/b included SPT2, AN32A, SET, NP1L4/NAP1L4, NPM1/NPM, and NUCL, and ASF1a also associated with NP1L1/NAP1L1 and NPM3. The histone-dependent association of ASF1 isoforms with Nap1-like proteins (NAP1L1 and NAP1L4) parallels the abilities of both yeast Nap1 and Vps75 to form complexes with Asf1–H3–H4,^12^ attesting that these Nap1-like proteins also bind H3–H4 dimers in mammals. NAP1L4 and NPM1/3 also formed co-chaperone complexes with DAXX and HJURP, respectively, positioning these multifunctional chaperones at the intersection between branches of the H3–H4 network. In line with this analysis, NPM1 has also been shown to associate with both non-nucleosomal H3–H4 and CENPA-H4.^40^^,^^41^ Finally, sNASP interacted with the histone reader UBR7 via histones, demonstrating the histone dependence of this recently reported interaction.^83^

In addition, we identified several histone-dependent interactions with chromatin remodelers (Figure 3A). DAXX had histone-dependent relationships with the chromatin remodelers CHD1 and the ChAHP complex (CHD4, ADNP, and CBX1/3). With the ability of DAXX to deposit H3.3–H4 with the chromatin remodeler ATRX, these links to other chromatin remodeling enzymes could allow H3.3–H4 deposition by DAXX at other genomic sites. In addition, the chromatin remodeler ERCC6/CSB (and its alternative splicing product, ERPG3) was identified in ASF1a/b and sNASP histone-dependent complexes. ERCC6 remodels chromatin during transcription-coupled nucleosome excision repair,^84^ and our result suggests ASF1 and sNASP assist in this process.

To demonstrate the resource potential of our network analysis, we validated several histone-dependent interactions (Figure 3B), including sNASP with UBR7, ASF1b with ERCC6 and SPT2, and DAXX with HP1y (CBX3), ADNP, and SUV39H1 (SUV91). We also found that ASF1b associates with ERCC6 and SPT2 independent of its B domain unlike CAF-1,^49^ and DAXX binding to ATRX contributes to the association with HP1y (CBX3) and ADNP but not SUV39H1 (SUV91) (Figure 3B). Using AlphaFold 2.0,^85^^,^^86^ we predicted the structure of the ASF1a–H3.1–H4–SPT2 co-chaperone complex (Figures S3A and S3B). From this, we identified an interacting region containing the histone-binding domains of SPT2, ASF1, and the histone fold of H3.1–H4, which generated high-confidence scores in both PAE and pLDDT metrics (Figures 3C, 3D, and S3A–S3C). Alignment of this structural prediction with the crystal structure of SPT2-(H3.2–H4)_2_ indicated that the SPT2 αC2 helix (Figures 3E, S3D, and S3E), normally associated with the H3–H4 tetramerization interface, is relocated to allow ASF1a to maintain its binding mode with H3–H4 (Figure S3E).^46^^,^^87^ To experimentally validate this prediction, we generated two SPT2 mutants corresponding to previously identified HBMs based on the yeast SPT2–H3–H4 complex^88^, M641E642AA (M1) also contacting histone H4 in the co-chaperone structure and E662D663AA (M2) located in the SPT2 αC2 helix and not predicted to bind histones. The interaction of SPT2 with histones and ASF1 was lost in the M1 mutant but not the M2 mutant (Figure S3F), supporting the predicted SPT2–ASF1 co-chaperone interaction.

### DAXX operates as an isolated arm of the histone H3–H4 chaperone network

**Figure 4:**
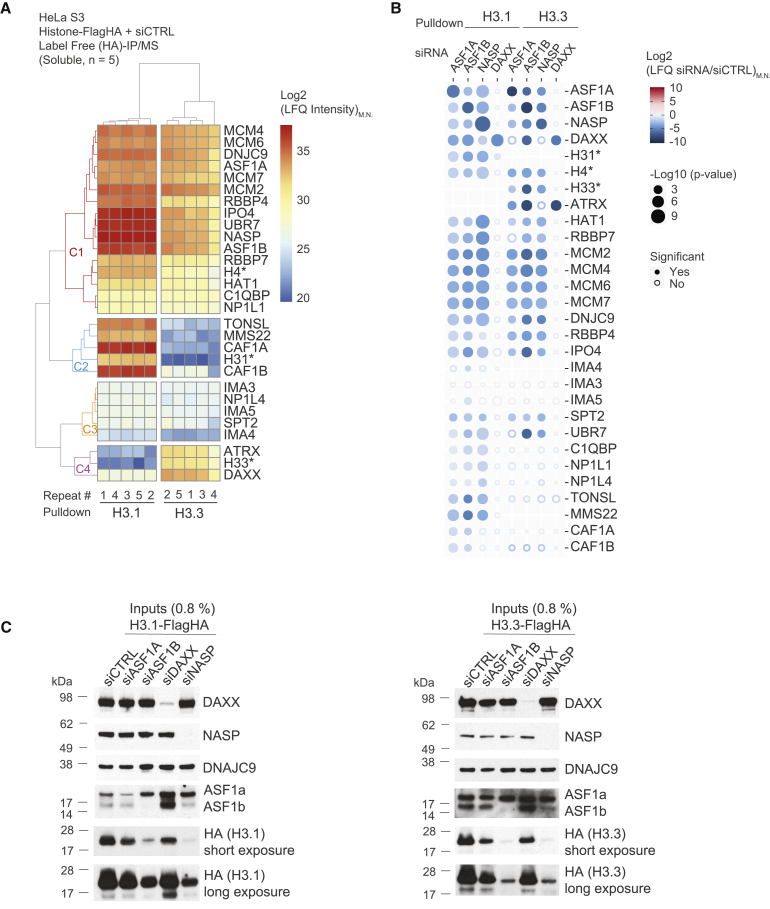
Histone chaperone perturbation demonstrates their functional connectivity (A) Clustering analysis showing Euclidean distances between median normalized LFQ intensities (LFQ_M.N._) for proteins identified in H3.1 and H3.3 pull-downs by MS. (B) Bubble plot showing the changes in abundance of proteins identified in histone H3.1 and H3.3 pull-downs from extracts siRNA depleted of the chaperones ASF1A, ASF1B, NASP, and DAXX compared with control conditions (siCTRL). Colors represent Log2 ratios of median normalized LFQ intensities (siRNA/siCTRL), and radii represent p values. (A and B) Representative of n = 5 biological replicates, with median normalized LFQ intensities quantified on a protein or ^∗^peptide level by MS. Proteins are referred to by human UniProt protein identification code. See also Table S1. (C) Western blot of soluble extracts from cells expressing H3.1-FlagHA (left) and H3.3-FlagHA (right) siRNA depleted for ASF1A, ASF1B, DAXX, or NASP and compared with control knockdowns siCTRL. Representative of n = 5 biological replicates. See also Figure S4.

To further dissect the role of ASF1a, ASF1b, NASP, and DAXX in the histone supply chain, we addressed how depletion of these chaperones influenced the interactomes of soluble H3.1 and H3.3, using label-free quantification (LFQ)-coupled MS analysis (Figures 4A–4C). We focused our analysis on well-known histone H3–H4 chaperones and their associated binding partners, several importins (IPO4, IMA3, IMA4, and IMA5), and some more recently implicated histone-binding factors (UBR7 and C1QBP).^69^^,^^83^

First, we performed hierarchical clustering analysis of LFQ intensities for these proteins compared with the peptide-level LFQ intensities for H3.1, H3.3, and H4, to track the abundance of proteins across experiments (Figure 4A). The intensity of histone H4 peptides was similarly distributed across the H3.1 and H3.3 pull-downs serving as a proxy for factors that bind H3–H4 independently of histone H3 isoform (Figure 4A, cluster C1). We observed that the histone chaperones ASF1a/b, DNAJC9, MCM2, RBBP4/7, NASP, and NAP1L1, along with MCM4/6/7, C1QBP, HAT1, IPO4, and UBR7 clustered together with histone H4, supporting a conserved function for these proteins in H3.1 and H3.3 supply pathways. Another cluster of proteins, which included IMA3, IMA4, IMA5, and NP1L4 and SPT2 had a similar enrichment profile across conditions (Figure 4A, cluster C3). However, their low abundance suggests that they play a more minor role in histone H3.1 and H3.3 supply. By contrast, DAXX and ATRX formed a distinct cluster with H3.3 (Figure 4A, cluster C4), and the TONSL-MMS22L complex was only consistently identified in H3.1 pull-downs and clustered with H3.1 peptides similarly to the CAF-1 complex (Figure 4A, cluster C2). This argues that TONSL is a H3.1 chaperone in humans, in line with recent findings with the plant homolog TONSUKU.^89^

We then compared the effects of chaperone knockdowns on these interactions (Figure 4B). NASP and ASF1 depletion led to a reduction of the soluble levels of exogenously expressed H3.1 and H3.3 (Figures 4B and 4C), with NASP and ASF1b having the strongest effect. Consistent with previous work,^16^ NASP depletion reduced the endogenous pool of soluble H3–H4 whereas ASF1a/b depletion did not (Figure S4). This is in line with ASF1a/b regulating soluble histone homeostasis mainly during excess of soluble histones, such as exogenous histone expression or replication stress.^68^ We envision that in this setting NASP and ASF1 collaborate to protect histones H3–H4 from degradation, as the histone binding mode of NASP involved in co-chaperoning histones with ASF1 is required for H3–H4 stability.^18^

Proteins that bind both H3.1 and H3.3 (Figure 4A, cluster C1), were in almost all cases consistently depleted under NASP, ASF1a, and ASF1b knockdown conditions (Figure 4B). This follows the levels of soluble histones in these conditions, underscoring that ASF1a/b and NASP are master regulators of histone H3.1–H4 and H3.3–H4 supply pathways. SPT2 followed the same trend (Figure 4B), confidently assigning its role in both H3.1 and H3.3 pathways. TONSL, MMS22/MMS22L, C1QBP, NP1L1, and NP1L4 were only lost in pull-downs where H3.1 levels were affected. This was also the case for CAF1A and CAF1B, although surprisingly not in the case of NASP depletion, demonstrating the critical link between ASF1 and the supply of histones H3.1–H4 to CAF-1. Meanwhile, DAXX depletion exclusively affected ATRX binding to histone H3.3, without impacting other histone chaperone associations. This argues that DAXX operates as a largely independent arm of the histone chaperone network when depositing histones to heterochromatin and contributes minimally to the supply of histones to other chaperone systems. Otherwise, DAXX and ATRX binding to H3.3 was dependent on ASF1a/b and particularly ASF1b, implying that ASF1b acts partially upstream of DAXX in H3.3 supply to heterochromatin.

### H3K9 trimethylation marks newly synthesized H3.3–H4 bound by DAXX

**Figure 5:**
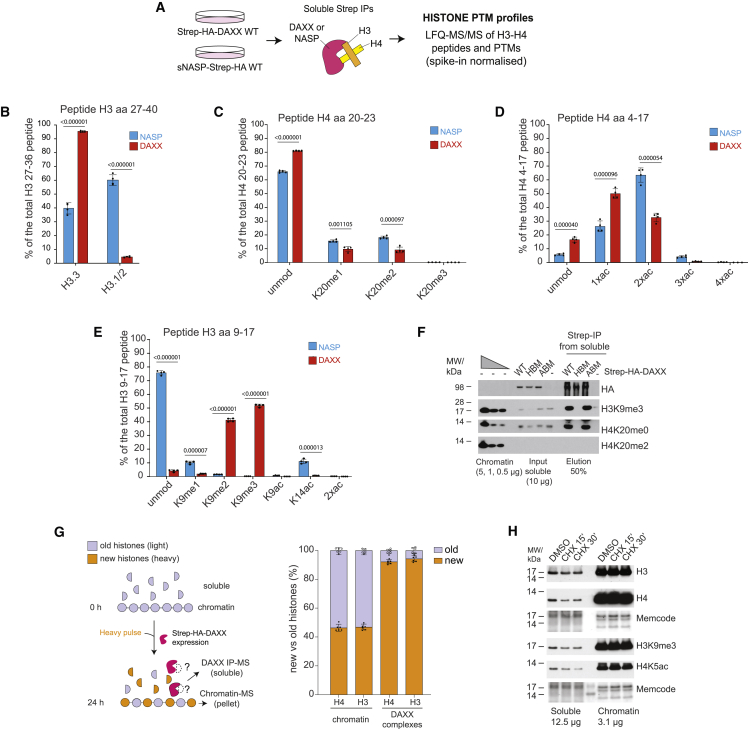
DAXX escorts new H3.3 K9 methylated histones prior to deposition (A) Strategy for profiling the peptides and PTMs of histones H3 and H4 from soluble sNASP and DAXX purifications. (B) Quantification of H3.1/2 and H3.3 peptides associated with sNASP and DAXX. (C–E) Quantification of PTMs on (C) H4 peptides 20–23, (D) H4 peptides 4–17, and (E) H3 peptides 9–17 associated with sNASP and DAXX. (B–E) Percentages are relative to the total intensity of related peptides and averaged across n = 4 biological replicates of sNASP and DAXX purifications. PTMs were normalized using heavy peptides standards. Error bars represent SD, p-values represent unpaired two-sided t tests. See also Figures S5 and S7 and Table S2. (F) Pull-downs of Strep-HA-tagged DAXX WT, HBM, or ABM compared with control purifications (−) from soluble cell extracts probed by western blot for histone modifications and compared with histone PTM levels on chromatin by serial dilution. Representative of n = 2 biological replicates. (G) (Left) Experimental design. Strep-HA-DAXX expressing cells were pulsed with heavy SILAC medium for 24 h. (Right) Proportion of new and old histones on chromatin and DAXX soluble complexes across n = 6 biological replicates. Error bars represent SD. See also Table S1. (H) Western blot of soluble and chromatin extracts from cells treated with cycloheximide (CHX, 15–30 mins) and compared with DMSO control. Representative of n = 2 biological replicates.

The concept of a nucleosome assembly pathway dedicated to *de novo* heterochromatin assembly was proposed upon the identification of soluble histone H3 methylated at lysine 9 (H3K9me1 and H3K9me2).^22^^,^^90^^,^^91^^,^^92^ However, it remains unexplored whether and how such pre-modified histones are targeted specifically to heterochromatin. Given the histone-dependent association of DAXX with multiple heterochromatin factors, including readers and writers of H3K9me3 (Figure 2A), we considered DAXX a likely candidate to mediate targeted silencing by depositing pre-modified histones. To explore this hypothesis, we profiled the PTMs of histone H3–H4 dimers bound to DAXX, and sNASP for comparison, using targeted and quantitative PTM MS analysis with heavy peptide spike-in normalization (Figure 5A).

As expected, DAXX almost exclusively interacted with H3.3,^26^^,^^27^^,^^45^ whereas sNASP bound both H3.1/2 and H3.3^20^ (Figure 5B). Both chaperones were associated with newly synthesized histone H4, identified by the absence of methylation on H4K20^11^ and di-acetylation of histone H4 (Figures 5C and 5D) catalyzed by HAT-1 complex during histone supply.^93^^,^^94^ sNASP associates with the HAT-1 complex (Figure 2A), and ∼65% of sNASP-bound H4 were di-acetylated. Since >95% of histone H4 in complex with ASF1b are di-acetylated,^22^ our data support that sNASP and the HAT-1 complex are upstream of ASF1 in the histone supply chain.^19^ H4 di-acetylation was lower in the DAXX complex (∼30%, Figure 5D), potentially reflecting DAXX association with histone deacetylase complex members SAP18 (Figure 1B) and HDAC1/2.^35^^,^^95^

Histone PTMs prevalent on nucleosomal histones,^90^ including H3K4, H3K27, H3K36, and H3K79 methylation (Figures S5A–S5C, Table S2), were not identified on either DAXX- or NASP-bound histones. Low levels of K14, K18, and K23 acetylation were found in both complexes, while H3K56ac was not detected (Figures 5C, S5D, and S5E), similar to ASF1-bound histones.^22^ Strikingly, ∼90% of histone H3 in complex with DAXX was di- or tri-methylated on H3K9, while sNASP mainly associated with histones unmethylated at H3K9 (Figure 5E) similar to ASF1b.^22^ Consolidating these findings, we validated that DAXX associates with histones marked with H3K9me3, H4K20me0, and H4K5ac (Figures 5F and S5F). Moreover, we find that H3K9me3 modified histones are chaperoned specifically by DAXX, not NASP, in mouse embryonic stem cells (mESCs) (Figure S5G), corroborating an earlier observation of H3K9me3 on DAXX-bound histones in mESCs.^29^

These results argue that a pool of newly synthesized non-nucleosomal histones H3.3–H4 are modified by H3K9me3 and destined for deposition by DAXX in mouse and humans. To further demonstrate that DAXX associates with newly synthesized histones, as implied by the presence of H4K20me0, and not histones released from chromatin, we pulse-labeled newly synthesized proteins with heavy SILAC media and compared the heavy/light ratio of DAXX-bound and chromatin-associated histones (Figure 5G). DAXX-bound histones were highly enriched for new histones (∼92% heavy) in contrast to nucleosomal histones (∼45% heavy) (Figure 5G). Consistent with this, short term inhibition of protein translation, known to deplete the new histone pool,^96^^,^^97^ also reduced H3K9me3 levels in the soluble fraction (Figure 5H). Collectively, this demonstrates that DAXX chaperones newly synthesized histone H3.3–H4 carrying H3K9me3 prior to deposition on DNA, identifying a conserved DAXX-centered pathway for *de novo* heterochromatin assembly.

### DAXX stimulates H3K9me3 methyltransferase activity

**Figure 6:**
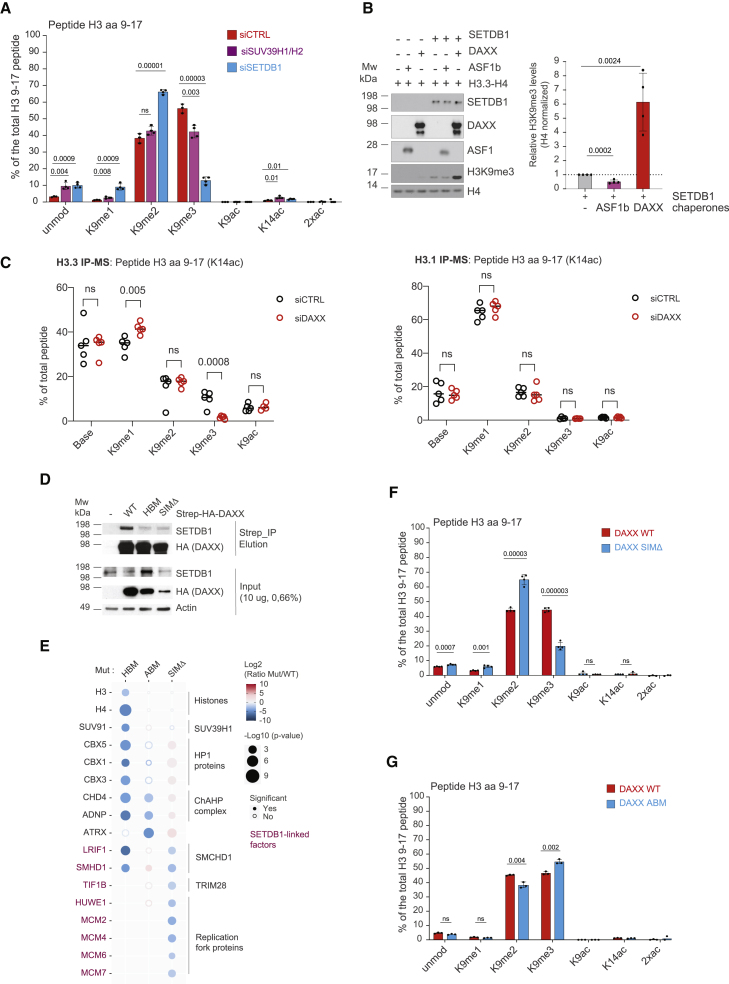
DAXX promotes the catalysis of H3.3 K9me3 in collaboration with SETDB1 (A) Quantification of PTMs on H3 peptides 9–17 associated with DAXX upon SETDB1, SUV39H1/H2, or control siRNA depletions averaged across n = 4 (siSETDB1 and siSUV39H1/H2) and n = 3 (siCTRL) biological replicates. See also Figures S6A and S7. (B) *In vitro* histone methyltransferase assay with recombinant proteins analyzed by western blot with average quantification of H3K9me3 representative of n = 4 independent experiments. H3K9me3 band intensities were normalized on corresponding H4 intensities. Error bars represent SD, p-values represent unpaired two-sided t tests. (C) LFQ quantification of K9 methylation on H3 peptides 9–17 acetylated on K14 detected in H3.3 (left) and H3.1(right) IP-MS. Mean values are indicated representative of n = 5 biological replicates, p-values represent unpaired two-sided t tests. Data from Figure 4 reanalyzed for PTMs, see STAR Methods. (D) Pull-downs of Strep-HA-tagged DAXX from soluble cell extracts expressing Strep-HA-DAXX WT, HBM, or SIMΔ mutants compared with control purifications probed by western blot. Images shown are representative of n = 2 biological replicates. See also Figure S6B. (E) Bubble plot showing the changes in abundance for factors associated with DAXX HBM, ABM, or SIMΔ mutants compared with WT DAXX. Proteins are referred to by human UniProt protein identification code. SETDB1-linked factors are indicated in magenta. Data generated from n = 4 biological replicates. DAXX WT vs. SIMΔ dataset was bait normalized to account for the lower expression level of DAXX SIMΔ mutant. Colors represent Log2 SILAC ratios (Mut/WT), and radii represent p values. See also Table S1 and Figures S6E and S6F. (F) Quantification of PTMs on H3 peptides 9–17 associated with DAXX WT or SIMΔ mutant averaged across n = 4 biological replicates. PTMs were normalized using heavy peptides standards. (G) Quantification of PTMs on H3 peptides 9–17 associated with DAXX WT or ABM mutant averaged across n = 3 biological replicates. (A, F, and G) Percentages are relative to the total intensity of related peptides and averaged across biological replicates of DAXX purifications. Error bars represent SD. PTMs were normalized using heavy peptides standards, p-values represent unpaired two-sided t tests. See also Table S2.

To identify the methyltransferase(s) contributing to H3K9me3 in DAXX complexes, we profiled the PTMs of DAXX-bound histones after depletion of key H3K9 methyltransferases. Depletion of either SETDB1 or SUV39H1/2, previously shown to associate with DAXX,^30^^,^^35^ resulted in significantly lower levels of DAXX associated H3K9me3 (Figures 6A and S6A, Table S2), with SETDB1 having the strongest effect. As DAXX-bound histones H3.3–H4 dimers are both di- and tri-methylated at H3K9 (Figure 5E), and DAXX interacts with readers and writers of K9 methylation (Figures 1B and 2A), this implicates DAXX in the handling of histones during H3K9me3 catalysis. In support of this hypothesis, we found that DAXX potentiates the activity of SETDB1 toward K9 trimethylation of H3.3–H4 dimers *in vitro* in contrast to ASF1b (Figure 6B), supporting that DAXX has a unique ability to stimulate the catalysis of H3K9me3 on H3.3–H4 dimers.

We then revisited our H3.1 and H3.3 interactome data in DAXX-depleted conditions (Figure 4B) to assess effects on soluble H3K9 methylation levels. In these datasets, we detected H3K9me1/2/3 only on peptides containing H3K14ac, as these interactome samples were not propionylated prior to tryptic digestion in contrast to our targeted PTM analyses (Figure 6C). As H3K14ac has been observed to recruit SETDB1,^98^ this peptide is also biologically relevant for DAXX-mediated H3K9me3. While H3K9me3K14ac was largely absent on soluble H3.1, we observed ∼10% H3K9me3K14ac on soluble H3.3 (Figure 6C). Importantly, depletion of DAXX caused a significant reduction in H3.3 associated H3K9me3K14ac levels, mirrored by a significant increase in H3K9me1K14ac. Meanwhile, H3K9 methylation states on H3.1 remained stable upon DAXX depletion. This further supports that DAXX specifically promotes the catalysis of H3K9me3 on newly synthesized H3.3 in cells.

### DAXX–H3.3–H4 recruits SUV39H1/2 and SETDB1

SUV39H1/2 is a histone-dependent interactor of DAXX, but SETDB1 was not detected in DAXX interactomes (Figures 1 and 2). However, using less stringent conditions we captured the DAXX–SETDB1 interaction (Figures 6D and S6B), reported previously.^35^ Further, we found that the DAXX interaction with SETDB1 was also histone-dependent (Figures 6D and S6B). Considering that the association of DAXX with ATRX and SUMOylated proteins can drive DAXX-mediated transcriptional silencing,^34^^,^^35^^,^^99^ we addressed the role of ATRX and SUMOylation in methyltransferase recruitment and H3K9me3. For this, we generated two mutants based on previous studies,^35^^,^^100^ an ATRX-binding mutant (ABM) and a deletion mutant of the two annotated SUMO interacting motifs (SIMΔ) (Figures S6C and S6D). Comparison of the interactomes of DAXX ABM and SIMΔ mutants with the WT and HBM (Figures 6E, S6E, and S6F) demonstrated that the recruitment of SUV39H1 (SUV91) was strictly histone dependent and not influenced by ATRX or SUMOylation. Again, SETDB1 was not identified under IP-MS conditions, but lower stringency IPs revealed SETDB1 associates with DAXX in a SIM-dependent manner (Figures 6D and S6B). The DAXX–SETDB1 interaction is also not reliant upon ATRX.^35^ To summarize, both SUV39H1 and SETDB1 are histone-dependent interactors of DAXX, while SETDB1 recruitment also requires the association of DAXX with SUMOylated partner protein(s).

Further comparison of these interactomes showed that factors involved in heterochromatin silencing associate with DAXX with differential dependencies on ATRX and SUMOylation, while histone binding is not affected in either of the DAXX ABM and SIMΔ mutants (Figures 6E, S6E, and S6F). DAXX interacted with members of the ChAHP chromatin remodeling complex (ADNP and CHD4)^82^ in an ATRX-dependent manner (Figures 6E, 3B, and S6E), corroborating ATRX binding to ADNP.^33^ Finally, our results revealed that DAXX forms a substantial number of SUMO-dependent interactions (Figure S6F), likely reflecting the SUMO-dependent recruitment of DAXX to subnuclear compartments including PML bodies.^36^^,^^99^ This included LRIF1, SMCHD1 (SMHD1), TIF1B/TRIM28/KAP1, and replication fork proteins (MCM2-7 and HUWE1) (Figure 6E), all of which are annotated SUMOylated proteins^101^ and known or putative SETDB1-linked factors.^33^^,^^102^^,^^103^^,^^104^^,^^105^

**Figure 7:**
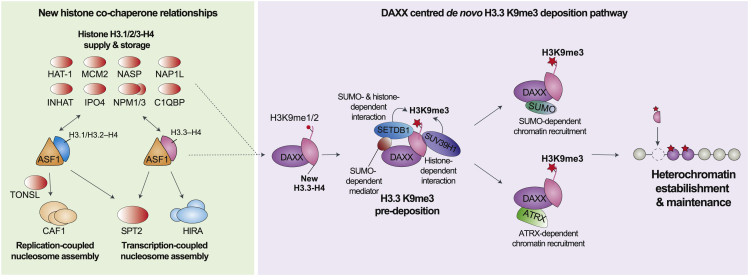
DAXX adds a *de novo* H3.3K9me3 deposition pathway to the histone chaperone network During histone supply ASF1 handles H3.1/2/3–H4 dimers and forms several histone-dependent co-chaperone complexes with other histone chaperones, notably ASF1 channels H3 variants toward distinct deposition complexes on chromatin. We identified a new ASF1-centered histone supply pathway to SPT2 that is H3 variant independent, as well as an upstream role for ASF1 in delivering H3.3 histones to DAXX, and an H3.1 variant specificity in TONSL. We found DAXX facilitates the catalysis of H3.3K9me3 through SETDB1 and SUV39H1 methyltransferase recruitment prior to histone deposition on chromatin. DAXX-bound H3.3–H4 recruits SETDB1 and SUV39H1, and the interaction of DAXX with SETDB1 is additionally dependent on SUMOylation. Other factors involved in heterochromatin establishment are differentially dependent on ATRX (e.g., the ChAHP complex) and SUMOylation (e.g., SMCHD1-LRIF1), and we speculate that this represents alternative pathways for H3K9me3 deposition supporting *de novo* heterochromatin silencing at distinct genomic locations.

Next, we profiled the PTMs of histones associated with the DAXX SIMΔ and ABM mutants (Figures 6F and S6H, Table S2). Strikingly, the DAXX SIMΔ mutant reduced H3K9me3 levels associated with DAXX to a similar extent as the loss observed upon depletion of SETDB1 (Figure 6, Figure 6A and 6F). Consistent with this, treatment with the SUMO-activating-enzyme inhibitor ML-792^106^ also reduced H3K9me3 on DAXX-bound histones (Figure S6H). Meanwhile, the loss of ATRX caused a minor increase in the levels of H3K9me3 with DAXX (Figure 6G, Table S2) not detectable by western blotting (Figure 5F). Collectively, these results demonstrate that the ability of DAXX to stimulate H3K9me3 of soluble histones is dependent on SUMOylation but does not depend on ATRX. Meanwhile, both ATRX and SUMO binding coordinate DAXX interactions with discrete heterochromatin complexes (Figure 6E), potentially orchestrating *de novo* H3.3 K9me3 deposition at alternative chromatin sites (Figure 7).

## Discussion

In this work, we provide a comprehensive interactome analysis of the histone chaperones central to H3 variant supply pathways. We discover unexplored histone-dependent connections with other cellular processes and reveal unique functionalities contributed by each chaperone to the global histone chaperone network. In addition, we identify a mechanism for targeted *de novo* heterochromatin formation through dissection of the DAXX assembly pathway. This strengthens our understanding of histone chaperone biology as a whole and reveal that nucleosome assembly pathways can be dedicated to deposition of modified histones and the formation of specialized chromatin states.

We expand the central role of ASF1 in histone supply, identifying uncharacterized histone-dependent co-chaperone complexes with SPT2, C1QBP, NPM1-3, and NAP1L1/4 proteins (Figure 7). We demonstrate how the histone-binding mode of SPT2, previously observed in association with a (H3–H4)_2_ tetramer,^88^ is remodeled to facilitate formation of the co-chaperone complex with ASF1. This is reminiscent of MCM2, Vps75, and Nap1, which can all bind (H3–H4)_2_ tetramers alone and form co-chaperone complexes with ASF1 and dimeric H3–H4.^9^^,^^12^^,^^107^ SPT2 and HIRA are both linked to transcription,^27^^,^^88^^,^^108^ but while HIRA is H3.3 specific,^20^ our data reveal that SPT2 binds both H3.1 and H3.3. Given that *de novo* H3.1–H4 is not deposited during transcription,^20^ we envisage that ASF1-SPT2 cooperation could be important during transcription-coupled histone recycling, which implicates ASF1 in both yeast^109^ and mammals.^110^

Our interrogation of the histone chaperone network also revealed a H3.1 specificity of TONSL-MMS22L, which has important implications for the mechanism of marking post-replicated chromatin for error-free DNA repair. In this respect, TONSL would read H4 K20me0 to specify new histones^11^ and H3.1 to be delivered to chromatin at sites of DNA replication (Figure 7). This may be required to restrict TONSL-MMS22L function in homologous recombination to post-replicative chromatin, as replication-independent deposition of new H3.3–H4 carrying H4K20me0 would not support TONSL-MMS22L recruitment. Notably, the plant homolog TONSUKU is also specific for H3.1^89^ but lacks the ankyrin repeat domain required for H4K20me0 recognition and a MMS22L plant homolog has yet to be identified.^11^^,^^89^ Together, this suggests that TONSL-MMS22L has post-replicative functions not mirrored by a plant counterpart.

We identified DAXX as an independent arm of the histone chaperone network that stimulates catalysis of H3K9me3 on H3.3–H4 dimers prior to their deposition onto DNA. We show that DAXX promotes the methyltransferase activity of SETDB1 toward H3K9me3. However, while DAXX-bound H3K9me3 levels are significantly reduced by SETDB1 and SUV39H1/2 depletion, we did not observe a complete loss of H3 K9me1/2/3. The strong accumulation of H3K9me2 in DAXX complexes upon SETDB1 depletion but not SUV39H1/H2 depletion supports that SETDB1 plays a major role in the conversion of H3K9me2 to H3K9me3. These differences could be indicative of a sequential pathway for H3K9me3 establishment in which SUV39H1/2 and SETDB1 act in a partially redundant manner. SETDB1 is also responsible for the catalysis of K9me1/2 on a fraction of new histone H3 during translation^92^ and H3K9me1/2 marking of new histones has previously been proposed to potentiate heterochromatin assembly.^14^^,^^90^^,^^91^ Notably ∼5% of ASF1b-bound histones carry H3K9me1 in S phase^22^ that may represent H3.3 destined for the DAXX-centered *de novo* H3.3K9me3 deposition pathway. Consistent with this notion, we found that the supply of histones H3.3–H4 to DAXX has a dependency on ASF1b, and a similar collaborative link has been identified between the DAXX-like protein (DLP) and ASF1 in flies.^111^

While SUV39H1 forms a stable histone-dependent interaction with DAXX, SETDB1 recruitment to DAXX–H3.3–H4 is more transient and mediated both by histones and SUMOylation, potentially via bridging factors, e.g., TRIM28 and PML bodies (Figure 7). In this respect, auto-SUMOylation of TRIM28 is important for its association with SETDB1,^112^^,^^113^ and both DAXX and SETDB1 localizes to PML bodies in a SUMO-dependent manner.^36^^,^^99^^,^^114^^,^^115^ Future structural studies will be required to address the molecular details of how DAXX orchestrates H3K9me3 catalysis, however, the significant reorientation of the H3 αN helix observed in the DAXX complexes^45^ might position the H3 tail for H3K9me3 catalysis by SETDB1.

Complexes with the potential to recruit DAXX-bound H3.3–H4 show differential dependencies on ATRX and SUMOylation, which is likely required for H3K9me3 deposition at distinct chromatin locations (this work and previous work^35^). ATRX associates with heterochromatin and is a H3K9me3 reader,^116^^,^^117^ therefore the collaboration of ATRX with DAXX–H3.3–H4 likely represents a H3K9me3 “read and deposit” mechanism for heterochromatin silencing. ATRX is also required for interaction of DAXX–H3.3–H4 with the ChAHP chromatin remodeling complex, which could target ADNP-binding sites for H3K9me3-mediated silencing. Consistent with this model, ADNP-binding sites in euchromatin show moderate enrichment for H3K9me3.^82^ Meanwhile, loss of SUMO binding prevents DAXX interaction with SMCHD1/SMHD1, and its binding partner LRIF1. SMCHD1 is a known reader of H3K9me3, which functions in X chromosome and repeat silencing.^78^^,^^79^^,^^81^^,^^105^^,^^118^ We thus propose that targeting DAXX–H3.3–H4 to different regions of the genome provides a mechanism to direct *de novo* assembly of H3K9me3 marked heterochromatin (Figure 7). This nucleosome assembly pathway could both support other silencing systems (e.g., H3K9me3 read-write, DNA methylation, and sequence-dependent histone methyltransferase recruitment) and act as a seed for heterochromatin formation.

The deposition of H3.3 by DAXX is required for heterochromatic silencing of diverse DNA templates including repetitive DNA elements, imprinted regions,^29^^,^^30^^,^^31^ and viral genomes after infection.^119^^,^^120^^,^^121^ However, H3.3 is also deposited by the HIRA complex at sites of active transcription.^20^^,^^27^ Our study resolves why DAXX and H3.3 are required for heterochromatin silencing because DAXX orchestrates H3K9me3 catalysis on H3.3–H4 prior to deposition. We envisage that this DAXX functionality supports heterochromatin maintenance counter-acting the dilution of H3K9me3 during DNA replication and allows the *de novo* assembly of heterochromatin, e.g., on viral genomes.

### Limitations of the study

Our proteomic analysis integrates interactome datasets from seven histone H3–H4 chaperones, however additional H3–H4 histones chaperones exist that were not directly targeted in our analysis. Our work should therefore serve as a framework that can be further expanded in the future, and to promote this, we have included our interactive Cytoscape networks as supplementary material (Data S1). Other approaches, such as proximity labeling proteomics (Bio-ID or APEX2) may also help to capture transient interactions our experimental strategy may have missed. It is possible that some of the identified interactions are mediated by RNA or other proteins, therefore reconstitution-based and structural approaches will help to resolve details of the provided interactomes. Finally, structural investigations and *in vitro* reconstitution will be important to reveal mechanistically how DAXX chaperoning of H3.3–H4 stimulates SETDB1 catalysis of H3K9me3.

## STAR★Methods

### Key resources table

**Table:** 

REAGENT or RESOURCE	SOURCE	IDENTIFIER
**Antibodies**		

H3K9me3	Abcam	Cat# ab176916, RRID:AB_2797591
H3K9me0	Active Motif	Cat# 91155, RRID:AB_2793790
DAXX	Sigma	HPA008736, RRID:AB_1078625
NASP	Abcam	Cat# ab181169
ASF1	Groth et al^68^	N/A
HA-tag	Cell Signaling Technology	Cat# 5017, RRID:AB_10693385
H4K20me0	Abcam	Cat# ab227804
H4K20me2	Diagenode	Cat# C15200205, RRID:AB_2877177
DNAJC9	Abcam	Cat# ab150394, RRID:AB_2890229
Tubulin	Abcam	Cat# ab6160, RRID:AB_305328
Actin	Sigma	Cat# A5316, RRID:AB_476743
SUV39H1	Cell Signaling Technology	Cat# 8729, RRID:AB_10829612
SUV39H2	Abcam	ab107225, RRID:AB_10861045
SETDB1/ESET	Abcam	Cat# ab107225, RRID:AB_10861045
SETDB1/ESET	ProteinTech	Cat# 11231-1-AP RRID:AB_2186069
H3	Abcam	Cat# ab10799, RRID:AB_470239
H4	Abcam	Cat# ab17036, RRID:AB_1209245
H3.1/2	Sigma	Cat# ABE154 RRID:AB_2811170
Mouse IgG2a	Abcam	Cat# ab18415, RRID:AB_2722535
Rabbit IgG	Cell Signaling Technology	Cat# 2729, RRID:AB_1031062
SPT2	Abcam	Cat# ab215722
UBR7	Bethyl	Cat# A304-130A RRID:AB_2621379
CBX3/HP1y	Abcam	Cat# ab56978 RRID: AB_941917
ADNP	Bethyl	Cat# A300-104A-M RRID:AB_2779012
ERCC6/ERPG3	Santa Crutz	Cat#sc-166042 RRID:AB_2293445
Flag	Sigma	Cat# F7425 RRID:AB_439687
H4K5ac	Abcam	Cat# ab51997 RRID:AB_2264109

**Chemicals, peptides, and recombinant proteins**		

Doxycycline	Clontech	Cat# 631311
Sequencing Grade Modified Trypsin	Promega	Cat# V5113
Benzonase Nuclease	Millipore	Cat# 70746
ML-792	Medchem Express	Cat# HY-108702
SETDB1-Flag	Active Motif	Cat# 31452
DAXX-Myc-Flag	Origene	Cat# TP326603
(H3.3–H4)_2_ Tetramer	Reaction Biology	Cat# HMT-14-438
ASF1b-HIS tag	abcam	Cat# ab130033
S-adenosylmethionine (SAM)	Bionordika	Cat# NEB-B9003S
TCEP	Merck	Cat# 646547
IAA	Thermo Scientific	Cat# A39271
DMSO	Sigma	Cat# D2650
Lipofectamine 2000	Thermo Scientific	Cat# 11668019
GlutaMAX	GIBCO	Cat# 35050061
Non-essential amino acids	GIBCO	Cat# 11140050
Penicillin & streptomycin	GIBCO	Cat# 15140122
Puromycin	Sigma	Cat# 8833
Pierce™ 660nm Protein Assay Reagent	Thermo Scientific	Cat# 2660

**Critical commercial assays**		

Pierce™ Reversible Protein Stain Kit for Nitrocellulose Membranes	Thermo Scientific	Cat# 24580
Lenti-X GoStix Plus	Clontech	Cat# 631280

**Deposited data**		

Raw Mass Spectrometry Data Sets	This study	PRIDE accession codes: PXD034924 PXD034888 PXD038263
Uncropped blots	This study	Mendeley Data: https://doi.org/10.17632/pdbxxhy5xt.1

**Experimental models: Cell lines**		

HeLa S3	ATCC	Cat# CCL-2.2 RRID:CVCL_0058
HeLa S3 pLVX-TetOne-puro H3.1-FlagHA	Hammond et al^13^	N/A
HeLa S3 pLVX-TetOne-puro H3.3-FlagHA	Hammond et al^13^	N/A
HeLa S3 pLVX-TetOne-puro TwinStrep-HA (control cells)	Bao et al^18^	N/A
HeLa S3 pLVX-TetOne-puro TwinStrep-HA-ASF1a (Strep-HA-ASF1a-WT)	This study	N/A
HeLa S3 pLVX-TetOne-puro TwinStrep-HA-ASF1a-V94R (Strep-HA-ASF1a-HBM)	This study	N/A
HeLa S3 pLVX-TetOne-puro ASF1b-TwinStrep-HA (ASF1b-Strep-HA WT)	This study	N/A
HeLa S3 pLVX-TetOne-puro TwinStrep-HA-ASF1b (Strep-HA-ASF1b-WT)	This study	N/A
HeLa S3 pLVX-TetOne-puro TwinStrep-HA- ASF1b-D36A-D37A (Strep-HA-ASF1b-BDM)	This study	N/A
HeLa S3 pLVX-TetOne-puro sNASP-TwinStrep-HA (sNASP-WT-Strep-HA)	This study	N/A
HeLa S3 pLVX-TetOne-puro sNASP-E211A-E215A-E217A-E246A-Y249S-L252S-TwinStrep-HA (sNASP-HBM-Strep-HA)	This study	N/A
HeLa S3 pLVX-TetOne-puro TwinStrep-HA- HJURP (HJURP-Strep-HA-WT)	This study	N/A
HeLa S3 pLVX-TetOne-puro TwinStrep-HA- HJURP-F29A-Y40A-W66A (Strep-HA-HJURP-HBM)	This study	N/A
HeLa S3 pLVX-TetOne-puro TwinStrep-HA-DAXX (Strep-HA-DAXX-WT)	This study	N/A
HeLa S3 pLVX-TetOne-puro TwinStrep-HA- DAXX-Y222A-R251A-R328A (Strep-HA-DAXX-HBM)	This study	N/A
HeLa S3 pLVX-TetOne-puro TwinStrep-HA- DAXX-F87A-Y124A (Strep-HA-DAXX-ABM)	This study	N/A
HeLa S3 pLVX-TetOne-puro TwinStrep-HA-DAXX-1/6Δ-734/740Δ (Strep-HA-DAXX-SIMΔ)	This study	N/A
Mouse: E14 ES cells	Laboratories of Kristian Helin and Joshua Brickman	RRID:CVCL_C320
293FT	Thermo Scientific	Cat# R70007, RRID: CVCL_6911

**Oligonucleotides**		

Silencer® Select Negative Control No. 1	Thermo Scientific	Cat# 4390843
Silencer® Select SETDB1 siRNA	Thermo Scientific	Cat# 138242
Silencer® Select SUV39H1 siRNA	Thermo Scientific	Cat# s13658
Silencer® Select SUV39H2 siRNA	Thermo Scientific	Cat# s36183
Silencer® Select NASP siRNA	Thermo Scientific	Cat# s9282
Silencer® Select DAXX siRNA	Thermo Scientific	Cat# s3937
Silencer® Select ASF1a siRNA	Thermo Scientific	Cat# s24602
Silencer® Select ASF1b siRNA	Thermo Scientific	Cat# s31346

**Recombinant DNA**		

pLVX-TetOne-puro	Bao et al^18^	N/A
pLVX-TetOne-puro TwinStrep-HA	Bao et al^18^	N/A
pLVX-TetOne-puro sNASP-TwinStrep-HA (sNASP-WT-Strep-HA)	Bao et al^18^	N/A
pLVX-TetOne-puro sNASP-E211A-E215A-E217A-E246A-Y249S-L252S-TwinStrep-HA (sNASP-HBM-Strep-HA)	This study	N/A
pLVX-TetOne-puro TwinStrep-HA-ASF1a (Strep-HA-ASF1a-WT)	This study	N/A
pLVX-TetOne-puro TwinStrep-HA-ASF1a-V94R (Strep-HA-ASF1a-HBM)	This study	N/A
pLVX-TetOne-puro TwinStrep-HA-ASF1b (Strep-HA-ASF1b-WT)	This study	N/A
pLVX-TetOne-puro TwinStrep-HA- ASF1b-V94R (Strep-HA-ASF1b-HBM)	This study	N/A
pLVX-TetOne-puro TwinStrep-HA- ASF1b-D36A-D37A (Strep-HA-ASF1b-BDM)	This study	N/A
pLVX-TetOne-puro TwinStrep-HA- HJURP(HJURP-Strep-HA-WT)	This study	N/A
pLVX-TetOne-puro TwinStrep-HA- HJURP-F29A-Y40A-W66A (Strep-HA-HJURP-HBM)	This study	N/A
pLVX-TetOne-puro TwinStrep-HA-DAXX (Strep-HA-DAXX-WT)	This study	N/A
pLVX-TetOne-puro TwinStrep-HA- DAXX-Y222A-R251A-R328A (Strep-HA-DAXX-HBM)	This study	N/A
pLVX-TetOne-puro TwinStrep-HA- DAXX-F87A-Y124A (Strep-HA-DAXX-ABM)	This study	N/A
pLVX-TetOne-puro TwinStrep-HA-DAXX-1/6Δ-734/740Δ (Strep-HA-DAXX-SIMΔ)	This study	N/A
pLVX-TetOne-puro H3.1-FlagHA	Hammond et al^13^	N/A
pLVX-TetOne-puro H3.3-FlagHA	Hammond et al^13^	N/A
pCMV6-ASF1a-Myc-Flag (WT)	Origene	Cat# RC200324
pCMV6-ASF1b- Myc-Flag (WT)	Origene	Cat# RC206114
pCMV6-sNASP-Myc-Flag (WT)	Origene	Cat# RC208783
pCMV6-HJURP-Myc-Flag (WT)	Origene	Cat# RC201283
pCMV6-DAXX-Myc-Flag (WT)	Origene	Cat# RC226603
pCMV6-SPTY2D1-Myc-Flag (WT)	Origene	Cat# RC223508
pCMV6-SPTY2D1-M641A-E642A-Myc-Flag (M1)	This study	N/A
pCMV6-SPTY2D1-E662A-D663A-Myc-Flag (M2)	This study	N/A
pcDNA5-Empty	Thermo Scientific	V103320
pVSV	Addgene	Cat# 138479 RRID:Addgene_138479
psPAX2	Addgene	Cat# 12260 RRID:Addgene_12260

**Software and algorithms**		

MaxQuant 1.6.3.4	(Cox and Mann, 2008, Cox et al., 2011)	https://maxquant.net/maxquant/
Perseus 1.6.14.0	(Tyanova et al., 2016)	https://maxquant.net/perseus/
GraphPad Prism v9	GraphPad Software	https://www.graphpad.com/scientific-software/prism/
Cytoscape 3.9.1	(Shannon et al., 2003)	https://cytoscape.org/
stringApp 1.7.0	(Doncheva et al., 2019)	http://apps.cytoscape.org/
Omics Visualizer app 1.3.0	(Legeay et al., 2020)	http://apps.cytoscape.org/
ClusterMaker2 app 2.0	(Morris et al., 2011)	http://apps.cytoscape.org/
R Studio 1.4.1717	The R Foundation	https://cran.r-project.org/
PyMOL	The PyMOL Molecular Graphics System, Version 1.7, Schrodinger, LLC	https://www.pymol.org/2/
ImageJ 1.0	(Schindelin et al., 2012)	https://imagej.nih.gov/ij/download.html
Alpha Fold v2.0	Evans et al^85^, Jumper et al^86^	https://github.com/deepmind/alphafold

**Other**		

anti-HA beads	Thermo Scientific	Cat# 26181
MagStrep "type3" XT beads	Iba	Cat# 2-4090-010
BXT buffer	Iba	Cat# 2-1042-025
Protein A Agarose beads	Thermo Scientific	Cat# 20333
Anti-Flag agarose beads	Millipore	Cat# A2220

### Resource availability

#### Lead Contact

Further information and requests for resources and reagents should be directed to and will be fulfilled by the Lead Contact, Anja Groth (anja.groth@cpr.ku.dk).

#### Materials Availability

All stable and unique reagents generated in this study are available from the Lead Contact subject to a Materials Transfer Agreement.

### Experimental model and subject details

#### Cell line generation and transfection

H3.1 (HeLa S3 pLVX-TetOne-puro H3.1-FlagHA), H3.3 (HeLa S3 pLVX-TetOne-puro H3.3-FlagHA) and control (HeLa S3 pLVX-TetOne-puro-TwinStrep-HA) cell lines were published previously – see Key Resources Table. Cell lines expressing ASF1a, ASF1b, sNASP, HJURP and DAXX (WT and mutants) from pLVX-TetOne-puro constructs were created by lentiviral transductions of HeLa S3 suspension cells followed by 24 hrs of selection in puromycin (1 μg/ml). The lentivirus-containing media were collected and filtered using a 0.45 μm syringe 60 hrs after transfecting of 293FT cells with 5 μg pVSV, 8 ug psPAX2 and 10 μg pLVX-TetOne plasmids using Lipofectamine 2000 reagents according to the manufacturer’s instructions. The presence of lentiviral particles was confirmed using Lenti-X GoStix Plus according to the manufacturer’s instructions. All used cell lines generated in this study tested negative for mycoplasma contamination. HeLa S3 and 293FT cell lines were derived from female subjects. SPT2 WT and mutants (M1 and M2) plasmids were transiently transfected for 24 hours in HeLa S3 cells expressing Strep-HA-ASF1b WT (1 ug/ml DOX, 24 hours) using Lipofectamine 2000 reagents according to the manufacturer’s instructions.

#### Cell culture

HeLa S3 and 293FT cells were grown in DMEM + GlutaMAX (Thermo Fisher Scientific) medium supplemented with 10 % FBS (Hyclone) and 1 % penicillin and streptomycin. E14 mESCs (male) were grown in on plates coated with 0.2 % gelatin (Sigma, G9391) in DMEM media (GIBCO, 10829018) supplemented with GlutaMAX-pyruvate (Thermo Fisher Scientific) with fetal bovine serum (15 %, Hyclone), LIF (made in-house), 1x non-essential amino acids (Gibco), 1x penicillin/streptomycin (Gibco) and 2-beta-mercaptoethanol (0.1 μM). E14 mESCs were passaged using Trypsin-EDTA (Gibco). All cells were grown in a humidified incubator at 37 °C with 5 % CO_2_. pLVX-TetOne-Puro HeLa S3 cell lines were generally grown under puromycin selection (1 μg/ml) and pulsed with doxycycline (Dox) to induce the expression of ASF1a, ASF1b, sNASP, HJURP, DAXX (2 μg/ml Dox, 24-36 hrs). Except cell lines expressing histones H3.1 and H3.3 which were induced with 100 ng/ml Dox for 48 hrs, after 48 hrs of siRNA depletion, following the protocol established previously.^13^ For SILAC experiments cells were grown in RPMI 1640 Medium for SILAC (Thermo scientific, 88365) supplemented with dialyzed FBS (Thermo Scientific), MEM non-essential amino acid mix (Thermo Scientific), GlutaMax (Thermo Scientific), and isotopically labelled arginine (316 μM) and lysine (547 μM). Triple SILAC experimental conditions employed heavy Lys8-Arg10, medium Lys4-Arg6, or light Lys0-Arg0. The HeLa S3 pLVX-TetOne-puro-TwinStrep-HA control cell line for all triple SILAC experiments was cultured with light amino acids Arg0 and Lys0 (A6969 and L8662, Sigma) in all biological replicates, while the HeLa S3 expressing WT and mutant chaperones were label-swapped between medium Arg6 and Lys4 (CNLM-2265-H1 and DLM-2640-1, Cambridge Isotope Laboratories) and heavy Arg10 and Lys8 (CNLM-539-H1 and CNLM-291-H-1, Cambridge Isotope Laboratories) amino acid pairs across biological replicates. For SILAC pulse experiments, cells were cultured DMEM medium with light amino acids Arg0 and Lys0 (A6969 and L8662, Sigma) and after 2 washes in PBS transferred to heavy SILAC medium (Arg10,Lys8) for 24 hrs prior to DAXX expression (1 μg/ml Dox, 22 hrs). Where indicated, cells were treated with 1 μM SUMO1/2 inhibitor ML-792^106^ for 6 hrs. For transient expression, cells were transfected with Lipofectamine 2000 and the plasmid DNA according to the manufacturer’s instructions.

### Method details

#### Plasmid generation

The TwinStrep-HA tag (sequence TGGGGSGGGASWSHPQFEKGGSGGGSWSH PQFEKGGYPYDVPDYA^∗^) was synthesised and cloned (by Genscript) between the EcoRI and BamHI sites of the pLVX-TetOne-Puro vector (631849, Clontech). ASF1a, ASF1b, sNASP, HJURP and DAXX cDNA were sub-cloned by amplifying their respective cDNA (from OriGene plasmids) with primers that create the homologous arms and then using these PCR products as “mega-primer” pairs to insert the gene product by site-directed mutagenesis into the pLVX-TetOne-puro-TwinStrep-HA construct. All the chaperone cDNAs were cloned in frame with an N-terminal of TwinStrep-HA tag, except for sNASP which was C-terminally TwinStrep-HA tagged. Mutations were introduced into coding sequences using established QuickChange mutagenesis protocols (Stratagene) or Infusion HD-directed mutagenesis (Takara). For the latter template plasmids were amplified with Phusion HF (F530S, Thermo Scientific) using mutagenic primers that also created homologous arms which, after PCR purification (28104, QIAgen) and Dpn1 digest (R0176L, NEB), were recombined through Infusion HD cloning (638933, Takara). DAXX 4HBΔ and SIMΔ mutant plasmids was prepared by Genscript.

#### Cell extracts

Soluble extracts were prepared by washing the cells twice with cold PBS and pelleting them by centrifugation (300 g, 3 mins) at 4 °C. The cell pellet was resuspended in ice cold NP40-NaCl buffer (300 mM NaCl, 0.05 % Nonidet P40, 50 mM Tris.HCl pH 7.6, 0.1 mM EDTA, 5 % glycerol) with freshly added inhibitors (NaF (5 mM) and β-Glycerolphosphate (10 mM), Phenylmethanesulfonyl fluoride (0.1 mM), Leupeptin (10 μg/ml), Pepstatin A (10 μg/ml), Trichostatin A (100 ng/ml), Na_3_VO_4_ (0.2 mM)) with 15 mins incubation at 4 °C. Subsequently, the lysate was cleared by centrifugation (11,000 g, 20 min), transferred to a new tube, centrifuged again (11,000 g, 10 mins) and filtered (0.45 μm). For analysis of the DAXX–SETDB1 interaction, soluble extracts were obtained by resuspending cell pellets in low salt version of the NP-40 NaCl buffer with 150 mM NaCl (instead of 300 mM) and otherwise processed as above. For chromatin extracts, the pellets derived from NP-40 NaCl soluble extraction (300 mM NaCl) were digested for 1 hour at 37 °C with 0.015 volumes of 25 U/μl Benzonase (Millipore, 70746) in 1 volume of the same buffer supplemented with 0.01 volumes of 1 M MgCl_2_. The resultant chromatin extracts were cleared by centrifugation (16,000 g, 3 min, 4 °C), and supernatants were transferred to new tubes. For SILAC pulse experiment, the chromatin extracts were prepared by washing the chromatin pellet with an additional 1 volume of NP-40 NaCl buffer prior to Benzonase digestion (0.015 volume, 25 U/ml, Millipore 70746, 2 hour at 37 °C) in NP-40 NaCl buffer supplemented with 10 mM MgCl_2_. For soluble vs chromatin fractionation extracts were prepared similarly but with the following modifications, soluble extracts were prepared with a modified NP-40 NaCl buffer (including 300 mM NaCl, 50 mM Hepes.KOH pH 7.9, 1 mM DTT, 200 ng/ml cycloheximide) and the chromatin pellet was washed with an additional 1 volume buffer prior to MNase digestion and resultant extracts were 0.45 μm spin filtered after centrifugation. Extracts were used directly for experiments or otherwise stored at −80 °C.

#### Immunoprecipitation and Western blot analysis

Protein concentrations were measured using Pierce™ 660 nm Protein Assay Reagent (Thermo Scientific) and equalized using NP40-NaCl extraction buffer. For immunoprecipitation of tagged proteins, Strep-HA-chaperone and histone-Flag-HA extracts were incubated with MagStrep "type3" XT beads (2-4090-010, IBA) or anti-HA (26181, Thermo scientific), respectively, for 3 hrs at 4 °C. After incubation, chaperone-IP beads were washed twice using ice-cold wash buffer (150 mM NaCl, 0.02 % Nonidet P40, 50 mM Tris.HCl pH 7.6, 0.1 mM EDTA, 5 % glycerol), and additionally washed four times with ice-cold wash buffer lacking glycerol and NP-40, prior to elution with 1X Strep-Tactin®XT elution buffer (BXT buffer, 2-1042-025, IBA) at RT for 1 h. SILAC-labeled samples were subjected to in-solution tryptic digestion, while samples were probed by Western blot analysis. Histone IPs from siRNA treated cell extracts were washed exclusively in NP40-NaCl buffer and then additionally washed with minimal wash buffer (MWB: 300 mM NaCl, 50 mM Tris.HCl pH 7.6) and NH_4_HCO_3_ (50 mM) prior to on-bead tryptic digestion as previously reported.^13^ Details of on-bead digestion are described in the “MS sample preparation” section. For immunoprecipitation of endogenous SETDB1, H3K9me0 and H3K9me3, 2-4 mg of soluble extracts were pre-cleared using 50 μL of BSA-blocked (10 mg/l BSA in 1.5 mM Tris.HCl, pH 7.6) Protein A-agarose beads (20333, Thermo Scientific) for 40 minutes at 4 °C, followed by incubation with 50 μl of antibody-coupled BSA-blocked beads (2 μg of H3K9me3, H3K9me0 or SETDB1 antibodies) for 4 hrs at 4 °C. Control rabbit IgG (2 μg, Cell Signaling Technology, 2729) and mouse IgG2a (2 μg, ab18415, Abcam) were coupled for the control pull-downs. The beads were then washed five times with ice-cold buffer (150 mM NaCl, 0.02 % Nonidet P40, 50 mM Tris.HCl pH 7.6, 0.1 mM EDTA, 5 % glycerol), and protein complexes were eluted using Laemmli sample buffer (50 mM Tris.HCl pH 6.8, 1 % SDS, 10 % glycerol, 25 mM DTT) for 20 minutes at 98 °C.

#### In vitro histone methyltransferases assay

100 nM of (H3.3–H4)_2_ tetramer (Reaction biology, HTM-14-438) were incubated with equimolar concentration of either recombinant DAXX (OriGene, TP326603) or ASF1b (Abcam, ab130033) in a reaction system containing 50 mM Tris.HCl pH 8, 0.02 % Triton X-100, 2 mM MgCl2, 1 mM TCEP, 50 mM NaCl, and 10 % glycerol, for 1 hour at RT. The reaction system was then supplemented with 2 nM of recombinant SEDTB1 (Active Motif, 3152) and 50 μM of SAM (Bionordika, NEB-B9003S) for 15 minutes at RT. H3K9me3 (dilution 1:1000, Abcam, ab176916) antibody was used in the Western blot analysis. Intensities of H3K9me3 bands were quantified relative to H4 in ImageJ version 1.0, and these relative intensities were normalized to the control condition lacking ASF1 and DAXX.

#### Antibodies

Western blots were performed with the following antibodies: DNAJC9 (1:1000, ab150394, Abcam), HA (1:3000-5000, C29F4 #3724, Cell Signaling Technology), DAXX (1:250, HPA008736, Sigma), H3 (1:500-1000, ab10799, Abcam), H4 (1:1000, ab17036, Abcam), Tubulin (1:10000, ab6160, Abcam), H3K9me3 (1:1000, ab194296, Abcam), H3K9me0 (1:1000, 91155, Active Motif), NASP (1:1000, ab181169, Abcam), H4K20me0 (1:500, ab227804, Abcam), H4K20me2 (1:500, C15200205, Diagenode), H4K5ac (1:1000, ab51997, Abcam), Actin (1:5000, A5316, Sigma), SUV39H1 (1:500, 8729, Cell Signaling Technology), SUV39H2 (1:500, ab107225, Abcam), SETDB1 (1:1000, ab107225, Abcam, IP 2 ug, 11231-1-AP, Proteintech), ASF1 (1:1000), ADNP (1:500, Bethyl), SPT2 (1:1000, Abcam), UBR7 (1:1000, Bethyl), CBX3 (1:500, ab56978, Abcam), ERCC6/ERPG3 (1:200 Santa Crutz), FLAG (1:1000, F7425, Sigma).

#### MS sample preparation

Interactome samples were trypsin digested either in-gel, in-solution, or on-beads. In-gel digestions were performed as previously reported,^122^ briefly, 15 μg of proteins per SILAC condition were mixed and loaded onto 4-12 % NuPAGE BIS-TRIS gels, and proteins were separated via electrophoresis. Gels were washed two hours in water, then gel bands were excised and cut into 1 mm^3^ cubes, and dehydrated using 100 % acetonitrile (ACN), followed by removal of ACN and brief drying using a vacuum centrifuge. Following this, dehydrated gel pieces were suspended in 50 mM ammonium bicarbonate containing 5 ng/μl of modified sequencing grade Trypsin (Sigma Aldrich), with just enough solution added to cover all gel pieces after expansion. Gel pieces were incubated overnight at 30 °C, after which two volumes of 0.1 % trifluoroacetic acid (TFA) in 30 % ACN were added. The supernatant was removed from the gel pieces and stored, and gel pieces were washed once more with two volumes of 0.1 % TFA in 30 % ACN, and finally washed with 100 % ACN, with all supernatants pooled together. The final supernatant was dried for 3 hours in a vacuum centrifuge at 60 °C (to evaporate all acetonitrile), prior to cleanup using C18 StageTips. For in-solution digested samples, cysteine residues were reduced and alkylated by concomitantly adding tris(2-carboxyethyl)phosphine (TCEP) pH 7.5 and chloroacetamide (CAA) to a final concentration of 5 mM for 30 min at 30 °C. Sample were then digested using 0.5 μg of modified sequencing grade Trypsin (Sigma Aldrich), overnight at 30 °C. On-bead digestions were performed by pre-washing beads 2x in ice-cold 50 mM ammonium bicarbonate, followed by resuspension of beads in two bead volumes of ice-cold 50 mM ammonium bicarbonate containing 5 ng/μl of modified sequencing grade Trypsin (Sigma Aldrich). Beads were kept mixed for 30 min to allow trypsin to diffuse, prior to moving beads to 30 °C and shaking them overnight. Following on-bead digestion, the supernatant containing peptides was separated from any beads via passage through 0.45 μm cutoff spin filters. Cysteine residues were reduced and alkylated by concomitantly adding TCEP and CAA to a final concentration of 5 mM for 30 min at 30 °C.

For all MS samples, peptides were desalted and purified using StageTips,^123^ using high-pH cleanup.^124^ Briefly, quad-layer StageTips were prepared using four punch-outs of C18 material (Sigma-Aldrich, Empore™ SPE Disks, C18, 47 mm). StageTips were equilibrated using 100 μl of methanol, 100 μl of 80 % ACN in 200 mM ammonium hydroxide, and two times 75 μl 50 mM ammonium. Samples were supplemented with 1/10^th^ volume of 200 mM ammonium hydroxide (pH >10), just prior to loading them on StageTip. The StageTips were subsequently washed twice with 150 μl 50 mM ammonium hydroxide, and afterwards eluted using 80 μl of 25 % ACN in 50 mM ammonium hydroxide. All fractions were dried to completion in protein-LoBind tubes (Eppendorf), using a SpeedVac for 2 h at 60°C, after which the dried peptides were dissolved using 11 μl of 0.1 % formic acid, and stored at −20 °C until MS analysis.

#### Sample preparation for histone modification analysis by MS

Sample preparation and MS analysis were performed according to the EpiQMAx GmbH protocols. Briefly, protein eluted from histone chaperones pulldowns were resuspended in Lämmli buffer and separated by a 14-20 % gradient SDS-PAGE, stained with Coomassie (Brilliant blue G-250). Protein bands in the molecular weight range of histones (15-23 kDa) were excised as single band/fraction. Gel slices were destained in 50 % acetonitrile/50mM ammonium bicarbonate. Heavy peptide standards were spiked in (166 fmoles each). Lysine residues were chemically modified by propionylation for 30 min at RT with 2.5 % propionic anhydride (Sigma) in ammonium bicarbonate, pH 7.5. Subsequently, proteins were digested with 200 ng of trypsin (Promega) in 50mM ammonium bicarbonate overnight and the supernatant was desalted by C18-Stagetips (reversed-phase resin) and carbon Top-Tips (Glygen) according to the manufacturer’s instructions. After desalting, the eluent was speed vacuumed until dryness and stored at -20°C until MS analysis.

#### MS analysis

The majority of MS samples were analyzed on an EASY-nLC 1200 system (Thermo) coupled to either a Q Exactive™ HF-X Hybrid Quadrupole-Orbitrap™ mass spectrometer (Thermo) or an Orbitrap Exploris™ 480 mass spectrometer (Thermo), respectively referred to as “HF-X” and “Exploris” hereafter. The exact hardware used for each MS raw data file is defined in the experimental design template available on ProteomeXchange (PXD034888). For each run, 1-5 μl of sample was injected. Separation of peptides was performed using 20-cm columns (75 μm internal diameter) packed in-house with ReproSil-Pur 120 C18-AQ 1.9 μm beads (Dr. Maisch). Elution of peptides from the column was achieved using a gradient ranging from buffer A (0.1 % formic acid) to buffer B (80 % acetonitrile in 0.1 % formic acid), at a flow of 250 nl/min. For HF-X runs, the gradient length was 100 min per sample, including ramp-up and wash-out, with an analytical gradient of 75 min ranging from 7 % B to 38 % B. For Exploris runs, the gradient length was 80 min per sample, including ramp-up and wash-out, with an analytical gradient of 57 min ranging from 7 % B to 30-36 % B depending on sample type (see experimental design template). Analytical columns were heated to 40°C using a column oven, and ionization was achieved using a Nanospray Flex Ion Source (Thermo) on the HF-X or a NanoSpray Flex™ NG ion source on the Exploris. Spray voltage set to 2 kV, ion transfer tube temperature to 275°C, and RF funnel level to 40 %. Full scan range was set to 300-1,500 *m*/*z* (HF-X) or 300-1,300 *m*/*z* (Exploris), MS1 resolution to 120,000, MS1 AGC target to 3,000,000 charges (HF-X) or 2,000,000 charges (Exploris), and MS1 maximum injection time to 120 ms (HF-X) or “Auto” (Exploris). Precursors with charges 2-6 were selected for fragmentation using an isolation width of 1.3 *m*/*z*, and fragmented using higher-energy collision disassociation (HCD) with normalized collision energy of 25. Precursors were excluded from re-sequencing by setting a dynamic exclusion of 100 s (HF-X) or 80 s (Exploris). MS2 resolution was set to 45,000, MS2 AGC target to 200,000 charges, minimum MS2 AGC target to 20,000 (HF-X) or intensity threshold to 230,000 charges per second (Exploris), MS2 maximum injection time to 90 ms (HF-X) or “Auto” (Exploris), and TopN to 9. Exceptions to MS2-specific parameters are listed in the experimental design template at PXD034888 for the majority of experiments, and in the methods details at PXD038263 for the SILAC pulse chase experiment.

#### LC-MS analysis of histone modifications

Peptides were re-suspended in 17 μl of 0.1 % TFA. A total of 5.0 μl were injected into a nano-HPLC device (Thermo Fisher Scientific) using a gradient from 4 % B to 90 % B (solvent A 0.1 % FA in water, solvent B 80 % ACN, 0.1 % FA in water) over 90 min at a flow rate of 300 nl/min in a C18 UHPCL column (Thermo Fisher Scientific). Data was acquired in PRM positive mode using a Q Exactive™ HF Hybrid Quadrupole-Orbitrap™ (Thermo Fisher Scientific) to identify and quantify specific N-terminal peptides of histone H3 and histone H4 proteins and their PTMs. MS1 spectra were acquired in the m/z range 250-1600 with resolution 60,000 at m/z 400 (AGC target of 3x10^6^). MS2 spectra were acquired with resolution 15,000 to a target value of 2x10^5^, maximum IT 60ms, isolation 2 window 0.7 m/z and fragmented at 27 % normalized collision energy. Typical mass spectrometric conditions were: spray voltage, 1.5kV, no sheath and auxiliary gas flow, heated capillary temperature, 250°C.

### Quantification and statistical analysis

#### Analysis of MS data

Triple SILAC chaperones IPs and histones IPs MS RAW data were analyzed using the version v1.6.3.4. Distinct experiments were analyzed in separate computational runs, as defined in the experimental design template available on ProteomeXchange (PXD034888 and PXD038263). The human FASTA database used in this study was downloaded from UniProt on May 13^th^, 2019. Default MaxQuant settings were used, with exceptions specified below. Label-free quantification was enabled for all sample. Matching between runs and second peptide search were enabled. For SILAC samples, the re-quantify option was activated and multiplicity was set to 2 (SILAC labels Arg0,Lys0 [light] and Arg10,Lys8 [heavy]) or 3 (SILAC labels Arg0,Lys0 [light] and Arg6,Lys4 [medium] and Arg10,Lys8 [heavy]), for double and triple SILAC samples respectively.

#### MS data analysis and quantification of histone modifications

Raw files were searched with the Skyline software version 21.1^125^ against histone H3 and H4 peptides and their respective PTMs with a precursor mass tolerance of 5 ppm. The chromatogram boundaries of +1, +2, +3 and +4 charged peaks were validated and the Total Area MS1 under the first 4 isotopomers was extracted and used for relative quantification and comparison between experimental groups. The Total Area MS1 of co-eluting isobaric peptides (i.e., H3K36me3 and H3K27me2K36me1) was resolved using their unique MS2 fragment ions. Relative abundances (percentages) were calculated as in the following example for H3K18 acetylation:

%H3K18ac = (H3K18ac_K23un + H3K18ac_K23ac) / (H3K18un_K23un + H3K18ac_K23unmod + H3K18un_K23ac + H3K18ac_K23ac) x 100 %, where "ac" indicates acetylation and "un" indicates unmodified.

#### Statistical analysis of MS data

For triple SILAC chaperones IPs, MS RAW MaxQuant outputs (proteinGroups.txt) were analyzed using the Perseus software, version 1.6.14.0. For all datasets, the proteomics data was filtered to exclude potential contaminants hits, reverse-database hits, and proteins identified via modified peptides only. For all datasets, except the triple SILAC ASF1a vs ASF1b vs control, the light, medium, and heavy SILAC channels were subjected to the LFQ algorithm to accurately quantify and normalize protein ratios as derived from the ratios of individual peptides.^126^ In proteinGroups.txt, these values are written as LFQ intensity L, M or H. While LFQ is an acronym for Label-Free Quantification, the algorithm also accurately normalizes the individual SILAC channels, respecting both inter- and intra-experiment values derived from the different labels. The LFQ-normalized SILAC channel values were log_2_-transformed and filtered for detection at n=4 in at least one experimental condition. Missing values were then imputed using the default Perseus setting (down shift of 1.8 and a width of 0.3). Student’s two-sample t testing was performed with permutation-based FDR control, with s0 and FDR values stated in Table S1 sheet for each experiment. For the triple SILAC ASF1a vs ASF1b vs control dataset, the LFQ-normalized SILAC channel values were log_2_-transformed and filtered for detection at n=2 in at least one experimental condition. See Table S1.

H3.1 and H3.3 datasets were analyzed using label-free mass spectrometry and data was processed, filtered and Log_2_ transformed similarly to triple SILAC datasets. The resultant matrix was then split into experiment type (H3.1 and H3.3), filtered for n=5/5 valid values in at least one siRNA or siCTRL condition, median-normalized and imputed. T-tests were then performed (S0=0.1, FDR=0.05) prior to merging the H3.1 and H3.3 matrices. The MaxQuant peptide quantification output (peptides.txt) was similarly processed to extract histone peptide LFQ intensities on which T tests were also performed (S0=0.1, FDR=0.05). See Table S1. For investigation of the H3K9 modification status within the H3.1 and H3.3 datasets in siDAXX and siCTRL conditions, MS raw files were re-analyzed with MaxQuant, with addition of lysine acetylation, lysine methylation, lysine dimethylation, and lysine trimethylation as variable modifications.

For histone post-translational modifications, Total Area MS1 values were corrected for technical variability based on the abundances of the SIL heavy standard peptides across the samples. SIL standards were spiked in each sample at the same concentration, therefore any variability observed on these heavy peptides must come from technical sources. The resulting heavy-normalized intensities of the endogenous PTMs were used to calculate relative abundances by grouping PTMs that occur on the same peptide sequence. Percentages were then compared between experimental groups using unpaired two-sided t-tests. See Table S2.

#### Data visualization and network analysis

Scatter and bar plot were visualized in GraphPad Prism, version 9.0. Bubble plots and heatmaps were visualized with R studio using the libraries ggplot2 version v3.3.3, scales version 1.1.1, RColorBrewer version 1.1.2, and pheatmap version 1.0.12. Network analysis was performed in Cytoscape version 3.9.1, with the stringApp version 1.7.0, the Omics Visualizer app version 1.3.0, ClusterMaker2 app version 2.0. Venn diagrams were generated in Cytoscape with the Venn and Euler Diagrams app version 1.0.3. Functionally associated histone-independent interactors (STRING score >0.6) and histone-dependent (string score>0.7) were clustered using Markov Clustering (MCL, granularity=3.5) in Cytoscape using the stringApp and ClusterMaker2. Ribosomal proteins, which are common contaminants, were excluded from network visualizations for clarity, but are listed in Table S1. All other statistical analysis was performed in GraphPad Prism version 9.0 and test details and p values are referred to in Figure legends.

#### Protein complex structure predictions

Structural predictions of SPT2-H3.1-H4-ASF1a were performed using AlphaFold v2.0^85^^,^^86^ in multimer mode with a maximum template date of 2021-11-01 and an input FASTA file of full-length protein sequences (UniProt IDs: P68431, P62805, Q9Y294 and Q68D10). The top five ranked models were similar in respect to the way SPT2 and ASF1 associated with H3.1–H4, and structural analysis of the highest confidence prediction, including PAE domain clustering analysis was performed using UCSF ChimeraX v1.4 (2022-04-07).^127^

## Data Availability

• Mass spectrometry datasets that support the findings have been deposited to the ProteomeXchange Consortium via the PRIDE partner repository (Perez-Riverol et al., 2019) with the accession codes: PXD034924 (Histone PTM datasets), PXD034888 (IP-MS datasets), PXD038263 (SILAC-pulse datasets). Raw data for western blots have been deposited at Mendeley Data: https://doi.org/10.17632/pdbxxhy5xt.1. • This paper does not report original code. • Any additional information required to reanalyze the data reported in this paper is available from the lead contact upon request.

## Supplemental information

Document S1. Figures S1–S7

Data S1. Cytoscape session file related to Figures 1B and 2A

Table S1. Statistically processed mass spectrometry data sets, related to Figures 1B, 1C, 2A, 2B, 4A, 4B, 5G, 6C, 6E, S1B–S1F, S2A, S2B, S6E, and S6F which support Figures 1, 2, and 6

Table S2. Histone PTM profiling datasets, related to Figures 5B–5E, 6A, 6F, 6G, and S5A–S5E which supports Figure 5

Document S2. Article plus supplemental information

## References

[1] Luger K., Mäder A.W., Richmond R.K., Sargent D.F., Richmond T.J. (1997). Crystal structure of the nucleosome core particle at 2.8 Å resolution. Nature.

[2] Lai W.K.M., Pugh B.F. (2017). Understanding nucleosome dynamics and their links to gene expression and DNA replication. Nat. Rev. Mol. Cell Biol..

[3] Nicetto D., Zaret K.S. (2019). Role of H3K9me3 heterochromatin in cell identity establishment and maintenance. Curr. Opin. Genet. Dev..

[4] Hammond C.M., Strømme C.B., Huang H., Patel D.J., Groth A. (2017). Histone chaperone networks shaping chromatin function. Nat. Rev. Mol. Cell Biol..

[5] Grover P., Asa J.S., Campos E.I. (2018). H3-H4 histone chaperone pathways. Annu. Rev. Genet..

[6] Pardal A.J., Fernandes-Duarte F., Bowman A.J. (2019). The histone chaperoning pathway: from ribosome to nucleosome. Essays Biochem..

[7] Stewart-Morgan K.R., Petryk N., Groth A. (2020). Chromatin replication and epigenetic cell memory. Nat. Cell Biol..

[8] Andrews A.J., Chen X., Zevin A., Stargell L.A., Luger K. (2010). The histone chaperone Nap1 promotes nucleosome assembly by eliminating nonnucleosomal histone DNA interactions. Mol. Cell.

[9] Huang H., Strømme C.B., Saredi G., Hödl M., Strandsby A., González-Aguilera C., Chen S., Groth A., Patel D.J. (2015). A unique binding mode enables MCM2 to chaperone histones H3-H4 at replication forks. Nat. Struct. Mol. Biol..

[10] Ricketts M.D., Frederick B., Hoff H., Tang Y., Schultz D.C., Singh Rai T., Grazia Vizioli M., Adams P.D., Marmorstein R. (2015). Ubinuclein-1 confers histone H3.3-specific-binding by the HIRA histone chaperone complex. Nat. Commun..

[11] Saredi G., Huang H., Hammond C.M., Alabert C., Bekker-Jensen S., Forne I., Reverón-Gómez N., Foster B.M., Mlejnkova L., Bartke T. (2016). H4K20me0 marks post-replicative chromatin and recruits the TONSL–MMS22L DNA repair complex. Nature.

[12] Hammond C.M., Sundaramoorthy R., Larance M., Lamond A., Stevens M.A., El-Mkami H., Norman D.G., Owen-Hughes T. (2016). The histone chaperone Vps75 forms multiple oligomeric assemblies capable of mediating exchange between histone H3-H4 tetramers and Asf1-H3-H4 complexes. Nucleic Acids Res..

[13] Hammond C.M., Bao H., Hendriks I.A., Carraro M., García-Nieto A., Liu Y., Reverón-Gómez N., Spanos C., Chen L., Rappsilber J. (2021). DNAJC9 integrates heat shock molecular chaperones into the histone chaperone network. Mol. Cell.

[14] Loyola A., Tagami H., Bonaldi T., Roche D., Quivy J.P., Imhof A., Nakatani Y., Dent S.Y.R., Almouzni G. (2009). The HP1α–CAF1–SetDB1-containing complex provides H3K9me1 for Suv39-mediated K9me3 in pericentric heterochromatin. EMBO Rep..

[15] Martire S., Banaszynski L.A. (2020). The roles of histone variants in fine-tuning chromatin organization and function. Nat. Rev. Mol. Cell Biol..

[16] Cook A.J.L., Gurard-Levin Z.A., Vassias I., Almouzni G. (2011). A specific function for the histone chaperone NASP to fine-tune a reservoir of soluble H3-H4 in the histone supply chain. Mol. Cell.

[17] Hormazabal J., Saavedra F., Espinoza-Arratia C., Martinez N.W., Cruces T., Alfaro I.E., Loyola A. (2022). Chaperone mediated autophagy contributes to the newly synthesized histones H3 and H4 quality control. Nucleic Acids Res..

[18] Bao H., Carraro M., Flury V., Liu Y., Luo M., Chen L., Groth A., Huang H. (2022). NASP maintains histone H3-H4 homeostasis through two distinct H3 binding modes. Nucleic Acids Res..

[19] Campos E.I., Fillingham J., Li G., Zheng H., Voigt P., Kuo W.H., Seepany H., Gao Z., Day L.A., Greenblatt J.F., Reinberg D. (2010). The program for processing newly synthesized histones H3.1 and H4. Nat. Struct. Mol. Biol..

[20] Tagami H., Ray-Gallet D., Almouzni G., Nakatani Y. (2004). Histone H3.1 and H3.3 complexes mediate nucleosome assembly pathways dependent or independent of DNA synthesis. Cell.

[21] Groth A., Corpet A., Cook A.J.L., Roche D., Bartek J., Lukas J., Almouzni G. (2007). Regulation of replication fork progression through histone supply and demand. Science.

[22] Jasencakova Z., Scharf A.N.D., Ask K., Corpet A., Imhof A., Almouzni G., Groth A. (2010). Replication stress interferes with histone recycling and predeposition marking of new histones. Mol. Cell.

[23] Gan H., Serra-Cardona A., Hua X., Zhou H., Labib K., Yu C., Zhang Z. (2018). The Mcm2-Ctf4-Polα axis facilitates parental histone H3-H4 transfer to lagging strands. Mol. Cell.

[24] Petryk N., Dalby M., Wenger A., Stromme C.B., Strandsby A., Andersson R., Groth A. (2018). MCM2 promotes symmetric inheritance of modified histones during DNA replication. Science.

[25] Nakamura K., Saredi G., Becker J.R., Foster B.M., Nguyen N.V., Beyer T.E., Cesa L.C., Faull P.A., Lukauskas S., Frimurer T. (2019). H4K20me0 recognition by BRCA1-BARD1 directs homologous recombination to sister chromatids. Nat. Cell Biol..

[26] Drané P., Ouararhni K., Depaux A., Shuaib M., Hamiche A. (2010). The death-associated protein DAXX is a novel histone chaperone involved in the replication-independent deposition of H3.3. Genes Dev..

[27] Goldberg A.D., Banaszynski L.A., Noh K.-M., Lewis P.W., Elsaesser S.J., Stadler S., Dewell S., Law M., Guo X., Li X. (2010). Distinct factors control histone variant H3.3 localization at specific genomic regions. Cell.

[28] Lewis P.W., Elsaesser S.J., Noh K.-M., Stadler S.C., Allis C.D. (2010). Daxx is an H3.3-specific histone chaperone and cooperates with ATRX in replication-independent chromatin assembly at telomeres. Proc. Natl. Acad. Sci. USA.

[29] Elsässer S.J., Noh K.-M., Diaz N., Allis C.D., Banaszynski L.A. (2015). Histone H3.3 is required for endogenous retroviral element silencing in embryonic stem cells. Nature.

[30] He Q., Kim H., Huang R., Lu W., Tang M., Shi F., Yang D., Zhang X., Huang J., Liu D., Songyang Z. (2015). The Daxx/Atrx complex protects tandem repetitive elements during DNA hypomethylation by promoting H3K9 trimethylation. Cell Stem Cell.

[31] Voon H.P.J., Hughes J.R., Rode C., De La Rosa-Velázquez I.A., Jenuwein T., Feil R., Higgs D.R., Gibbons R.J. (2015). ATRX plays a key role in maintaining silencing at interstitial heterochromatic loci and imprinted genes. Cell Rep..

[32] Tsai K., Cullen B.R. (2020). Epigenetic and epitranscriptomic regulation of viral replication. Nat. Rev. Microbiol..

[33] Teng Y.-C., Sundaresan A., O’Hara R., Gant V.U., Li M., Martire S., Warshaw J.N., Basu A., Banaszynski L.A. (2021). ATRX promotes heterochromatin formation to protect cells from G-quadruplex DNA-mediated stress. Nat. Commun..

[34] Dyer M.A., Qadeer Z.A., Valle-Garcia D., Bernstein E. (2017). ATRX and DAXX: mechanisms and mutations. Cold Spring Harb. Perspect. Med..

[35] Hoelper D., Huang H., Jain A.Y., Patel D.J., Lewis P.W. (2017). Structural and mechanistic insights into ATRX-dependent and -independent functions of the histone chaperone DAXX. Nat. Commun..

[36] Corpet A., Kleijwegt C., Roubille S., Juillard F., Jacquet K., Texier P., Lomonte P. (2020). PML nuclear bodies and chromatin dynamics: catch me if you can!. Nucleic Acids Res..

[37] Sadic D., Schmidt K., Groh S., Kondofersky I., Ellwart J., Fuchs C., Theis F.J., Schotta G. (2015). Atrx promotes heterochromatin formation at retrotransposons. EMBO Rep..

[38] Groh S., Milton A.V., Marinelli L.K., Sickinger C.V., Russo A., Bollig H., De Almeida G.P., Schmidt A., Forné I., Imhof A., Schotta G. (2021). Morc3 silences endogenous retroviruses by enabling Daxx-mediated histone H3.3 incorporation. Nat. Commun..

[39] Padeken J., Methot S.P., Gasser S.M. (2022). Establishment of H3K9-methylated heterochromatin and its functions in tissue differentiation and maintenance. Nat. Rev. Mol. Cell Biol..

[40] Dunleavy E.M., Roche D., Tagami H., Lacoste N., Ray-Gallet D., Nakamura Y., Daigo Y., Nakatani Y., Almouzni-Pettinotti G. (2009). HJURP is a cell-cycle-dependent maintenance and deposition factor of CENP-A at centromeres. Cell.

[41] Foltz D.R., Jansen L.E.T., Bailey A.O., Yates J.R., Bassett E.A., Wood S., Black B.E., Cleveland D.W. (2009). Centromere specific assembly of CENP-A nucleosomes is mediated by HJURP. Cell.

[42] Fujita Y., Hayashi T., Kiyomitsu T., Toyoda Y., Kokubu A., Obuse C., Yanagida M. (2007). Priming of centromere for CENP-A recruitment by human hMis18α, hMis18β, and M18BP1. Dev. Cell.

[43] Barnhart M.C., Kuich P.H.J.L., Stellfox M.E., Ward J.A., Bassett E.A., Black B.E., Foltz D.R. (2011). HJURP is a CENP-A chromatin assembly factor sufficient to form a functional de novo kinetochore. J. Cell Biol..

[44] Bowman A., Lercher L., Singh H.R., Zinne D., Timinszky G., Carlomagno T., Ladurner A.G. (2016). The histone chaperone sNASP binds a conserved peptide motif within the globular core of histone H3 through its TPR repeats. Nucleic Acids Res..

[45] Elsässer S.J., Huang H., Lewis P.W., Chin J.W., Allis C.D., Patel D.J. (2012). DAXX envelops a histone H3.3-H4 dimer for H3.3-specific recognition. Nature.

[46] English C.M., Adkins M.W., Carson J.J., Churchill M.E.A., Tyler J.K. (2006). Structural basis for the histone chaperone activity of Asf1. Cell.

[47] Hu H., Liu Y., Wang M., Fang J., Huang H., Yang N., Li Y., Wang J., Yao X., Shi Y. (2011). Structure of a CENP-A–histone H4 heterodimer in complex with chaperone HJURP. Genes Dev..

[48] Bowman A., Koide A., Goodman J.S., Colling M.E., Zinne D., Koide S., Ladurner A.G. (2017). sNASP and ASF1A function through both competitive and compatible modes of histone binding. Nucleic Acids Res..

[49] Tang Y., Poustovoitov M.V., Zhao K., Garfinkel M., Canutescu A., Dunbrack R., Adams P.D., Marmorstein R. (2006). Structure of a human ASF1a-HIRA complex and insights into specificity of histone chaperone complex assembly. Nat. Struct. Mol. Biol..

[50] Murachelli A.G., Ebert J., Basquin C., Le Hir H., Conti E. (2012). The structure of the ASAP core complex reveals the existence of a Pinin-containing PSAP complex. Nat. Struct. Mol. Biol..

[51] Hagerman R.J., Berry-Kravis E., Hazlett H.C., Bailey D.B., Moine H., Kooy R.F., Tassone F., Gantois I., Sonenberg N., Mandel J.L., Hagerman P.J. (2017). Fragile X syndrome. Nat. Rev. Dis. Primers.

[52] Kim Y.-E., Park C., Kim K.E., Kim K.K. (2018). Histone and RNA-binding protein interaction creates crosstalk network for regulation of alternative splicing. Biochem. Biophys. Res. Commun..

[53] Park J., Lee H., Han N., Kwak S., Lee H.-T., Kim J.-H., Kang K., Youn B.H., Yang J.-H., Jeong H.-J. (2018). Long non-coding RNA ChRO1 facilitates ATRX/DAXX-dependent H3.3 deposition for transcription-associated heterochromatin reorganization. Nucleic Acids Res..

[54] Pardal A.J., Bowman A.J. (2022). A specific role for importin-5 and NASP in the import and nuclear hand-off of monomeric H3. Elife.

[55] Apta-Smith M.J., Hernandez-Fernaud J.R., Bowman A.J. (2018). Evidence for the nuclear import of histones H3.1 and H4 as monomers. EMBO J..

[56] Tanaka K. (2009). The proteasome: overview of structure and functions. Proc. Jpn. Acad. Ser. B Phys. Biol. Sci..

[57] Collas P., Le Guellec K., Taskén K. (1999). The a-kinase–anchoring protein Akap95 is a multivalent protein with a key role in chromatin condensation at mitosis. J. Cell Biol..

[58] Magalska A., Schellhaus A.K., Moreno-Andrés D., Zanini F., Schooley A., Sachdev R., Schwarz H., Madlung J., Antonin W. (2014). RuvB-like ATPases function in chromatin decondensation at the end of mitosis. Dev. Cell.

[59] Yost S., de Wolf B., Hanks S., Zachariou A., Marcozzi C., Clarke M., de Voer R.M., Etemad B., Uijttewaal E., Ramsay E. (2017). Biallelic TRIP13 mutations predispose to Wilms tumor and chromosome missegregation. Nat. Genet..

[60] Müller S., Almouzni G. (2014). A network of players in H3 histone variant deposition and maintenance at centromeres. Biochim. Biophys. Acta.

[61] Prosser S.L., Pelletier L. (2017). Mitotic spindle assembly in animal cells: a fine balancing act. Nat. Rev. Mol. Cell Biol..

[62] Pan D., Walstein K., Take A., Bier D., Kaiser N., Musacchio A. (2019). Mechanism of centromere recruitment of the CENP-A chaperone HJURP and its implications for centromere licensing. Nat. Commun..

[63] Lee K.Y., Im J.-S., Shibata E., Dutta A. (2017). ASF1a promotes non-homologous end joining repair by facilitating phosphorylation of MDC1 by ATM at double-strand breaks. Mol. Cell.

[64] Yilmaz D., Furst A., Meaburn K., Lezaja A., Wen Y., Altmeyer M., Reina-San-Martin B., Soutoglou E. (2021). Activation of homologous recombination in G1 preserves centromeric integrity. Nature.

[65] Abascal F., Corpet A., Gurard-Levin Z.A., Juan D., Ochsenbein F., Rico D., Valencia A., Almouzni G. (2013). Subfunctionalization via adaptive evolution influenced by genomic context: the case of histone chaperones ASF1a and ASF1b. Mol. Biol. Evol..

[66] Tang M., Chen Z., Wang C., Feng X., Lee N., Huang M., Zhang H., Li S., Xiong Y., Chen J. (2022). Histone chaperone ASF1 acts with RIF1 to promote DNA end joining in BRCA1-deficient cells. J. Biol. Chem..

[67] Feng S., Ma S., Li K., Gao S., Ning S., Shang J., Guo R., Chen Y., Blumenfeld B., Simon I. (2022). RIF1-ASF1-mediated high-order chromatin structure safeguards genome integrity. Nat. Commun..

[68] Groth A., Ray-Gallet D., Quivy J.-P., Lukas J., Bartek J., Almouzni G. (2005). Human Asf1 regulates the flow of S phase histones during replicational stress. Mol. Cell.

[69] Lin J., Bao X., Li X.D. (2021). A tri-functional amino acid enables mapping of binding sites for posttranslational-modification-mediated protein-protein interactions. Mol. Cell.

[70] Okuwaki M., Kato K., Nagata K. (2010). Functional characterization of human nucleosome assembly protein 1-like proteins as histone chaperones. Genes Cells.

[71] Boer D.E.C., Van Smeden J., Bouwstra J.A., Aerts J.M.F.G. (2020). Glucocerebrosidase: functions in and beyond the lysosome. J. Clin. Med..

[72] Filmus J. (2001). Glypicans in growth control and cancer. Glycobiology.

[73] Pishesha N., Harmand T.J., Ploegh H.L. (2022). A guide to antigen processing and presentation. Nat. Rev. Immunol..

[74] Sánchez-Martín P., Saito T., Komatsu M. (2019). p62/SQSTM1: 'jack of all trades' in health and cancer. FEBS Journal.

[75] Chen R., Kang R., Fan X.G., Tang D. (2014). Release and activity of histone in diseases. Cell Death Dis..

[76] Silvestre-Roig C., Braster Q., Wichapong K., Lee E.Y., Teulon J.M., Berrebeh N., Winter J., Adrover J.M., Santos G.S., Froese A. (2019). Externalized histone H4 orchestrates chronic inflammation by inducing lytic cell death. Nature.

[77] Allshire R.C., Madhani H.D. (2018). Ten principles of heterochromatin formation and function. Nat. Rev. Mol. Cell Biol..

[78] Blewitt M.E., Gendrel A.V., Pang Z., Sparrow D.B., Whitelaw N., Craig J.M., Apedaile A., Hilton D.J., Dunwoodie S.L., Brockdorff N. (2008). SmcHD1, containing a structural-maintenance-of-chromosomes hinge domain, has a critical role in X inactivation. Nat. Genet..

[79] Brideau N.J., Coker H., Gendrel A.V., Siebert C.A., Bezstarosti K., Demmers J., Poot R.A., Nesterova T.B., Brockdorff N. (2015). Independent mechanisms target SMCHD1 to trimethylated histone H3 lysine 9-modified chromatin and the inactive X chromosome. Mol. Cell. Biol..

[80] Cebrià-Costa J.P., Pascual-Reguant L., Gonzalez-Perez A., Serra-Bardenys G., Querol J., Cosín M., Verde G., Cigliano R.A., Sanseverino W., Segura-Bayona S. (2020). LOXL2-mediated H3K4 oxidation reduces chromatin accessibility in triple-negative breast cancer cells. Oncogene.

[81] Gendrel A.V., Tang Y.A., Suzuki M., Godwin J., Nesterova T.B., Greally J.M., Heard E., Brockdorff N. (2013). Epigenetic functions of smchd1 repress gene clusters on the inactive X chromosome and on autosomes. Mol. Cell. Biol..

[82] Ostapcuk V., Mohn F., Carl S.H., Basters A., Hess D., Iesmantavicius V., Lampersberger L., Flemr M., Pandey A., Thomä N.H. (2018). Activity-dependent neuroprotective protein recruits HP1 and CHD4 to control lineage-specifying genes. Nature.

[83] Hogan A.K., Sathyan K.M., Willis A.B., Khurana S., Srivastava S., Zasadzińska E., Lee A.S., Bailey A.O., Gaynes M.N., Huang J. (2021). UBR7 acts as a histone chaperone for post-nucleosomal histone H3. EMBO J..

[84] Hargreaves D.C., Crabtree G.R. (2011). ATP-dependent chromatin remodeling: genetics, genomics and mechanisms. Cell Res..

[85] Evans R., O’Neill M., Pritzel A., Antropova N., Senior A., Green T., Žídek A., Bates R., Blackwell S., Yim J. (2021). Protein complex prediction with AlphaFold-multimer. Preprint at bioRxiv.

[86] Jumper J., Evans R., Pritzel A., Green T., Figurnov M., Ronneberger O., Tunyasuvunakool K., Bates R., Žídek A., Potapenko A. (2021). Highly accurate protein structure prediction with AlphaFold. Nature.

[87] Natsume R., Eitoku M., Akai Y., Sano N., Horikoshi M., Senda T. (2007). Structure and function of the histone chaperone CIA/ASF1 complexed with histones H3 and H4. Nature.

[88] Chen S., Rufiange A., Huang H., Rajashankar K.R., Nourani A., Patel D.J. (2015). Structure-function studies of histone H3/H4 tetramer maintenance during transcription by chaperone Spt2. Genes Dev..

[89] Davarinejad H., Huang Y.-C., Mermaz B., LeBlanc C., Poulet A., Thomson G., Joly V., Muñoz M., Arvanitis-Vigneault A., Valsakumar D. (2022). The histone H3.1 variant regulates TONSOKU-mediated DNA repair during replication. Science.

[90] Loyola A., Bonaldi T., Roche D., Imhof A., Almouzni G. (2006). PTMs on H3 variants before chromatin assembly potentiate their final epigenetic state. Mol. Cell.

[91] Pinheiro I., Margueron R., Shukeir N., Eisold M., Fritzsch C., Richter F., Mittler G., Genoud C., Goyama S., Kurokawa M. (2012). Prdm3 and Prdm16 are H3K9me1 methyltransferases required for mammalian heterochromatin integrity. Cell.

[92] Rivera C., Saavedra F., Alvarez F., Díaz-Celis C., Ugalde V., Li J., Forné I., Gurard-Levin Z.A., Almouzni G., Imhof A., Loyola A. (2015). Methylation of histone H3 lysine 9 occurs during translation. Nucleic Acids Res..

[93] Sobel R.E., Cook R.G., Perry C.A., Annunziato A.T., Allis C.D. (1995). Conservation of deposition-related acetylation sites in newly synthesized histones H3 and H4. Proc. Natl. Acad. Sci. USA.

[94] Verreault A., Kaufman P.D., Kobayashi R., Stillman B. (1998). Nucleosomal DNA regulates the core-histone-binding subunit of the human Hat1 acetyltransferase. Curr. Biol..

[95] Hollenbach A.D., McPherson C.J., Mientjes E.J., Iyengar R., Grosveld G. (2002). Daxx and histone deacetylase II associate with chromatin through an interaction with core histones and the chromatin-associated protein Dek. J. Cell Sci..

[96] Seale R.L., Simpson R.T. (1975). Effects of cycloheximide on chromatin biosynthesis. J. Mol. Biol..

[97] Mejlvang J., Feng Y., Alabert C., Neelsen K.J., Jasencakova Z., Zhao X., Lees M., Sandelin A., Pasero P., Lopes M., Groth A. (2014). New histone supply regulates replication fork speed and PCNA unloading. J. Cell Biol..

[98] Jurkowska R.Z., Qin S., Kungulovski G., Tempel W., Liu Y., Bashtrykov P., Stiefelmaier J., Jurkowski T.P., Kudithipudi S., Weirich S. (2017). H3K14ac is linked to methylation of H3K9 by the triple Tudor domain of SETDB1. Nat. Commun..

[99] Lin D.-Y., Huang Y.-S., Jeng J.-C., Kuo H.-Y., Chang C.-C., Chao T.-T., Ho C.-C., Chen Y.-C., Lin T.-P., Fang H.-I. (2006). Role of SUMO-interacting motif in Daxx SUMO modification, subnuclear localization, and repression of SUMOylated transcription factors. Mol. Cell.

[100] Escobar-Cabrera E., Okon M., Lau D.K.W., Dart C.F., Bonvin A.M.J.J., McIntosh L.P. (2011). Characterizing the N- and C-terminal small ubiquitin-like modifier (SUMO)-interacting motifs of the scaffold protein DAXX. J. Biol. Chem..

[101] Hendriks I.A., D'Souza R.C.J., Yang B., Verlaan-de Vries M., Mann M., Vertegaal A.C.O. (2014). Uncovering global SUMOylation signaling networks in a site-specific manner. Nat. Struct. Mol. Biol..

[102] Schultz D.C., Ayyanathan K., Negorev D., Maul G.G., Rauscher F.J. (2002). SETDB1: a novel KAP-1-associated histone H3, lysine 9-specific methyltransferase that contributes to HP1-mediated silencing of euchromatic genes by KRAB zinc-finger proteins. Genes Dev..

[103] Nozawa R.-S., Nagao K., Igami K.-T., Shibata S., Shirai N., Nozaki N., Sado T., Kimura H., Obuse C. (2013). Human inactive X chromosome is compacted through a PRC2-independent SMCHD1-HBiX1 pathway. Nat. Struct. Mol. Biol..

[104] Keniry A., Gearing L.J., Jansz N., Liu J., Holik A.Z., Hickey P.F., Kinkel S.A., Moore D.L., Breslin K., Chen K. (2016). Setdb1-mediated H3K9 methylation is enriched on the inactive X and plays a role in its epigenetic silencing. Epigenetics Chromatin.

[105] Ichihara S., Nagao K., Sakaguchi T., Obuse C., Sado T. (2022). SmcHD1 underlies the formation of H3K9me3 blocks on the inactive X chromosome in mice. Development.

[106] He X., Riceberg J., Soucy T., Koenig E., Minissale J., Gallery M., Bernard H., Yang X., Liao H., Rabino C. (2017). Probing the roles of SUMOylation in cancer cell biology by using a selective SAE inhibitor. Nat. Chem. Biol..

[107] Bowman A., Ward R., Wiechens N., Singh V., El-Mkami H., Norman D.G., Owen-Hughes T. (2011). The histone chaperones Nap1 and Vps75 bind histones H3 and H4 in a tetrameric conformation. Mol. Cell.

[108] Osakabe A., Tachiwana H., Takaku M., Hori T., Obuse C., Kimura H., Fukagawa T., Kurumizaka H. (2013). Vertebrate Spt2 is a novel nucleolar histone chaperone that assists in ribosomal DNA transcription. J. Cell Sci..

[109] Schwabish M.A., Struhl K. (2006). Asf1 mediates histone eviction and deposition during elongation by RNA polymerase II. Mol. Cell.

[110] Torné J., Ray-Gallet D., Boyarchuk E., Garnier M., Le Baccon P., Coulon A., Orsi G.A., Almouzni G. (2020). Two HIRA-dependent pathways mediate H3.3 de novo deposition and recycling during transcription. Nat. Struct. Mol. Biol..

[111] Fromental-Ramain C., Ramain P., Hamiche A. (2017). The Drosophila DAXX-Like Protein (DLP) cooperates with ASF1 for H3.3 deposition and heterochromatin formation. Mol. Cell. Biol..

[112] Ivanov A.V., Peng H., Yurchenko V., Yap K.L., Negorev D.G., Schultz D.C., Psulkowski E., Fredericks W.J., White D.E., Maul G.G. (2007). PhD domain-mediated E3 ligase activity directs intramolecular SUMOylation of an adjacent bromodomain required for gene silencing. Mol. Cell.

[113] Zeng L., Yap K.L., Ivanov A.V., Wang X., Mujtaba S., Plotnikova O., Rauscher F.J., Zhou M.-M. (2008). Structural insights into human KAP1 PhD finger-bromodomain and its role in gene silencing. Nat. Struct. Mol. Biol..

[114] Ishov A.M., Sotnikov A.G., Negorev D., Vladimirova O.V., Neff N., Kamitani T., Yeh E.T., Strauss J.F., Maul G.G. (1999). PML is critical for ND10 formation and recruits the PML-interacting protein Daxx to this nuclear structure when modified by SUMO-1. J. Cell Biol..

[115] Cho S., Park J.S., Kang Y.-K. (2011). Dual functions of histone-lysine N-methyltransferase Setdb1 protein at promyelocytic leukemia-nuclear body (PML-NB): maintaining PML-NB structure and regulating the expression of its associated genes. J. Biol. Chem..

[116] Dhayalan A., Tamas R., Bock I., Tattermusch A., Dimitrova E., Kudithipudi S., Ragozin S., Jeltsch A. (2011). The ATRX-ADD domain binds to H3 tail peptides and reads the combined methylation state of K4 and K9. Hum. Mol. Genet..

[117] Iwase S., Xiang B., Ghosh S., Ren T., Lewis P.W., Cochrane J.C., Allis C.D., Picketts D.J., Patel D.J., Li H., Shi Y. (2011). ATRX ADD domain links an atypical histone methylation recognition mechanism to human mental-retardation syndrome. Nat. Struct. Mol. Biol..

[118] Mould A.W., Pang Z., Pakusch M., Tonks I.D., Stark M., Carrie D., Mukhopadhyay P., Seidel A., Ellis J.J., Deakin J. (2013). Smchd1 regulates a subset of autosomal genes subject to monoallelic expression in addition to being critical for X inactivation. Epigenetics Chromatin.

[119] Tsai K., Chan L., Gibeault R., Conn K., Dheekollu J., Domsic J., Marmorstein R., Schang L.M., Lieberman P.M. (2014). Viral reprogramming of the Daxx histone H3.3 chaperone during early Epstein-Barr virus infection. J. Virol..

[120] Cliffe A.R., Knipe D.M. (2008). Herpes simplex virus ICP0 promotes both histone removal and acetylation on viral DNA during lytic infection. J. Virol..

[121] Cohen C., Corpet A., Roubille S., Maroui M.A., Poccardi N., Rousseau A., Kleijwegt C., Binda O., Texier P., Sawtell N. (2018). Promyelocytic leukemia (PML) nuclear bodies (NBs) induce latent/quiescent HSV-1 genomes chromatinization through a PML NB/histone H3.3/H3.3 Chaperone Axis. PLoS Pathog..

[122] Shevchenko A., Tomas H., Havlis J., Olsen J.V., Mann M. (2006). In-gel digestion for mass spectrometric characterization of proteins and proteomes. Nat. Protoc..

[123] Rappsilber J., Ishihama Y., Mann M. (2003). Stop and go extraction tips for matrix-assisted laser desorption/ionization, nanoelectrospray, and LC/MS sample pretreatment in proteomics. Anal. Chem..

[124] Hendriks I.A., Lyon D., Su D., Skotte N.H., Daniel J.A., Jensen L.J., Nielsen M.L. (2018). Site-specific characterization of endogenous SUMOylation across species and organs. Nat. Commun..

[125] Pino L.K., Searle B.C., Bollinger J.G., Nunn B., Maclean B., MacCoss M.J. (2020). The Skyline ecosystem: informatics for quantitative mass spectrometry proteomics. Mass Spectrom. Rev..

[126] Cox J., Hein M.Y., Luber C.A., Paron I., Nagaraj N., Mann M. (2014). Accurate proteome-wide label-free quantification by delayed normalization and maximal peptide ratio extraction, termed MaxLFQ. Mol. Cell. Proteomics.

[127] Pettersen E.F., Goddard T.D., Huang C.C., Meng E.C., Couch G.S., Croll T.I., Morris J.H., Ferrin T.E. (2021). UCSF ChimeraX: structure visualization for researchers, educators, and developers. Protein Sci..

