# Supplementary material for: DAXX adds a de novo H3.3K9me3 deposition pathway to the histone chaperone network
Source: Mol Cell. 2023 Apr 6;83(7):1075–1092.e9. doi: 10.1016/j.molcel.2023.02.009 (PMC10114496; doi:10.1016/j.molcel.2023.02.009)
Supplement: Document S1. Figures S1–S7 [file mmc1.pdf]

Molecular Cell, Volume *83*

Supplemental information

DAXX adds a *de novo* H3.3K9me3 deposition pathway to the histone chaperone network

Massimo Carraro, Ivo A. Hendriks, Colin M. Hammond, Victor Solis-Mezarino, Moritz Völker-Albert, Jonas D. Elsborg, Melanie B. Weisser, Christos Spanos, Guillermo Montoya, Juri Rappsilber, Axel Imhof, Michael L. Nielsen, and Anja Groth

SUPPLEMENTAL FIGURE TITLES AND LEGENDS

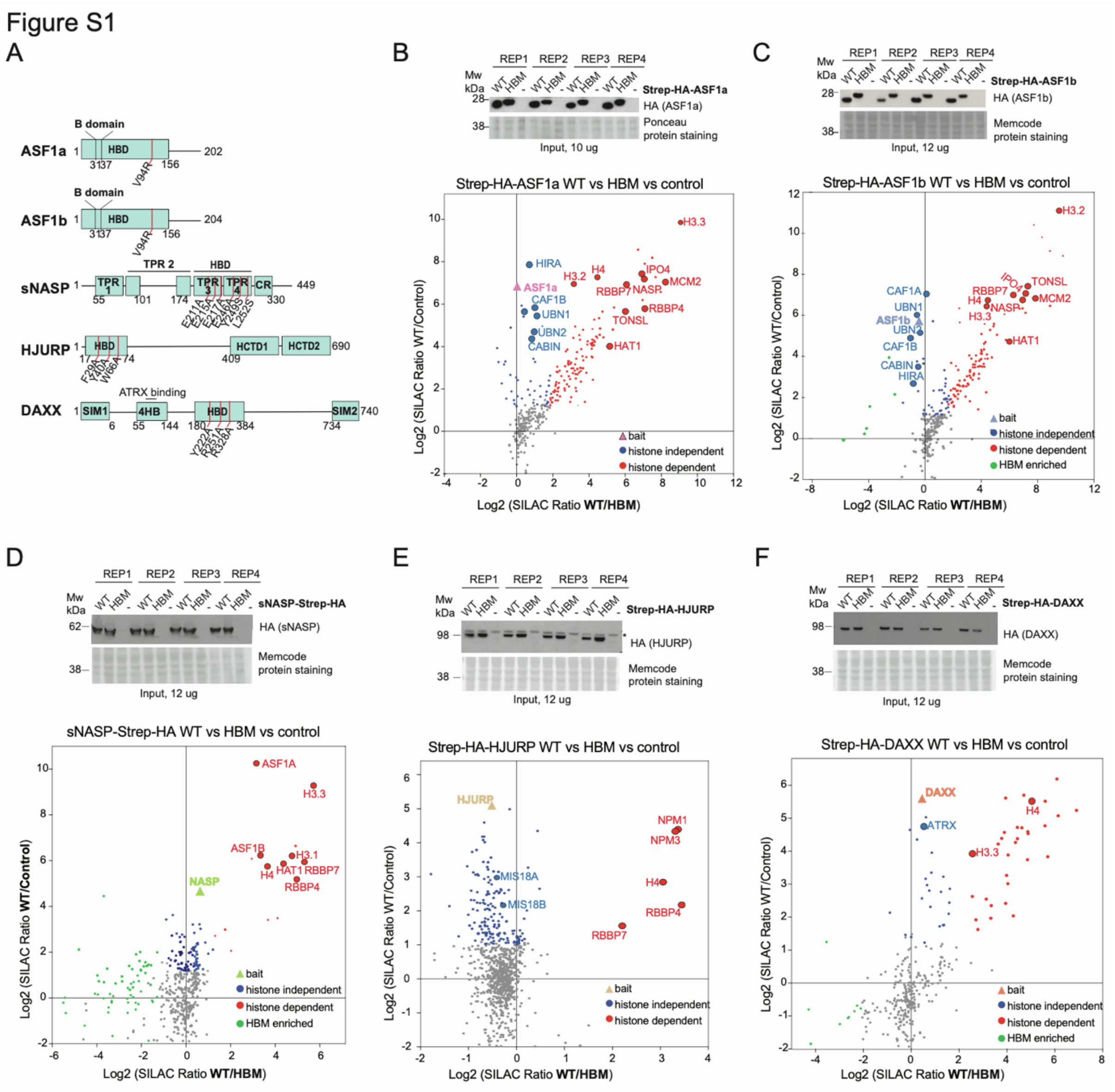

Figure S1. Input and IP-MS analysis of histone chaperones pulldowns – Related to Figures 1-2.
(A) Schematic domain architectures of ASF1a, ASF1b, sNASP, HJURP and DAXX. Red lines indicate the point mutations in the histone binding domain (HBD) used to disrupt the protein ability to bind histone H3–H4. (B-F) Top panels show Western blot analysis of soluble extracts from cells expressing STREP-HA-tagged ASF1a, ASF1b, sNASP, HJURP and DAXX (WT or HBM) and control cells (-), used to perform the triple SILAC IP/MS pulldowns shown in **Figures 1-2**. The panel show n=4 biological replicates. *, unspecific band. Bottom panels show mass spectrometry analysis of SILAC labelled pull-downs of ASF1a, ASF1b, sNASP, HJURP and DAXX, WT and HBM and control. Each pulldown was performed from soluble cell extracts, n=4 biological replicates. Proteins referred to by human UniProt protein identification code. The proteins nodes and names are colored according to the threshold indicated in **Table S1.** Red, blue, and green indicate histone-dependent, -independent, and HBM enriched factors, respectively.

Figure S2

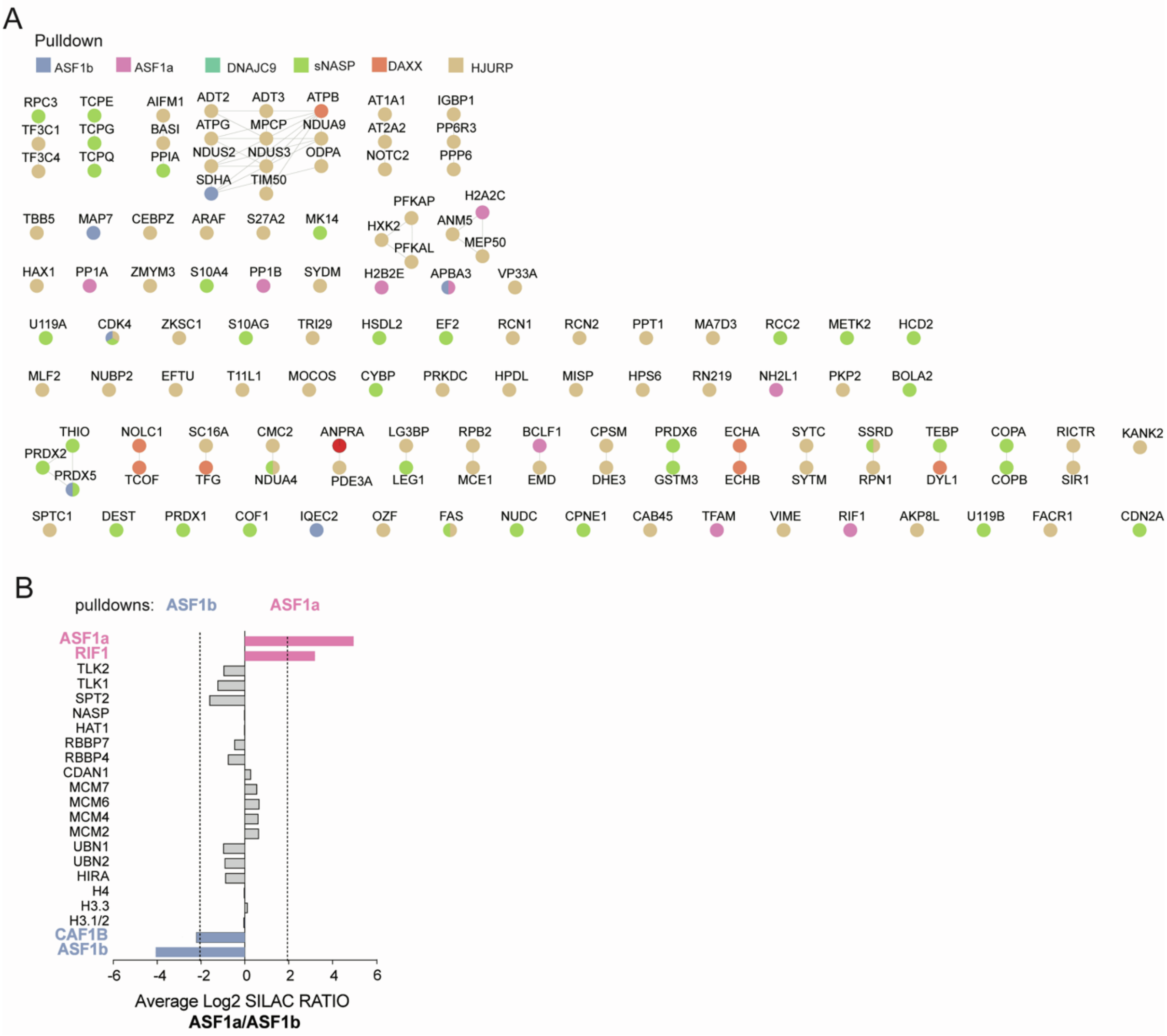

Figure S2. Analysis of histone-independent chaperone interactome – Related to Figure 1.

(A) Histone-independent interactors from clustered analysis not shown in Figure 1D, generated using the STRING database and MCL clustering function. Edges indicates Protein-protein interactions according to the string database.

(B) Mass spectrometry analysis of SILAC labelled pull-downs of wild type ASF1a and ASF1b and control cell from soluble cell extracts; n=2 biological replicates. Bar plots represent the average SILAC ratio.

(A-B) Proteins referred to by human UniProt protein identification code.

Figure S3

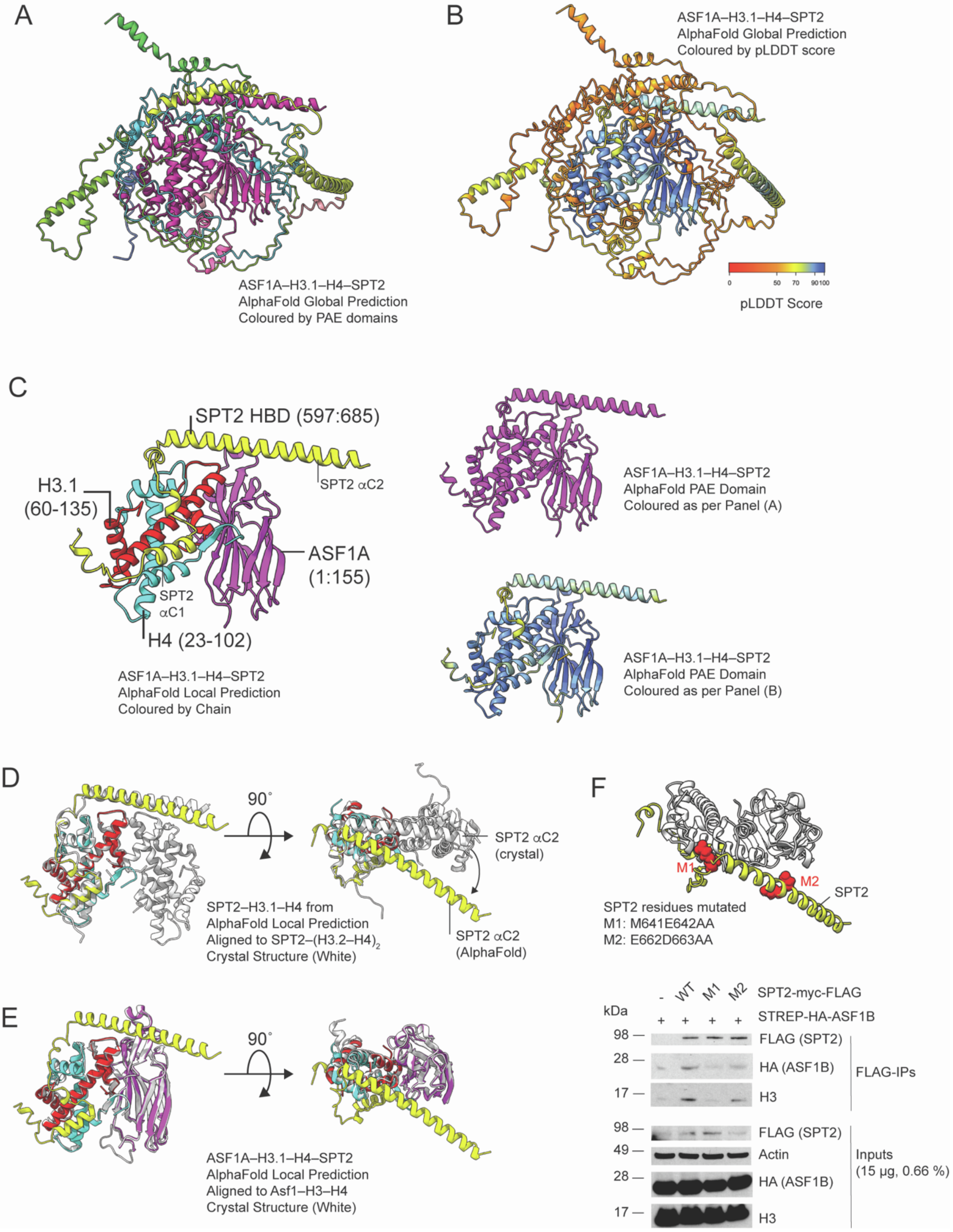

A
ASF1A–H3.1–H4–SPT2
AlphaFold Global Prediction
Coloured by PAE domains
B
ASF1A–H3.1–H4–SPT2
AlphaFold Global Prediction
Coloured by pLDDT score
0
50
70
90
100
pLDDT Score
C
SPT2 HBD (597:685)
H3.1
(60-135)
SPT2 αC2
ASF1A
(1:155)
SPT2
αC1
H4 (23-102)
ASF1A–H3.1–H4–SPT2
AlphaFold Local Prediction
Coloured by Chain
ASF1A–H3.1–H4–SPT2
AlphaFold PAE Domain
Coloured as per Panel (A)
ASF1A–H3.1–H4–SPT2
AlphaFold PAE Domain
Coloured as per Panel (B)
D
90°
SPT2 αC2
(crystal)
SPT2–H3.1–H4 from
AlphaFold Local Prediction
Aligned to SPT2–(H3.2–H4)2
Crystal Structure (White)
SPT2 αC2
(AlphaFold)
E
90°
ASF1A–H3.1–H4–SPT2
AlphaFold Local Prediction
Aligned to Asf1–H3–H4
Crystal Structure (White)
F
M1
M2
SPT2
SPT2 residues mutated
M1: M641E642AA
M2: E662D663AA
kDa
-
WT
M1
M2
SPT2-myc-FLAG
+
+
+
+
STREP-HA-ASF1B
98 —
FLAG (SPT2)
28 —
HA (ASF1B)
FLAG-IPs
17 —
H3
98 —
FLAG (SPT2)
49 —
Actin
Inputs
(15 µg, 0.66 %)
28 —
HA (ASF1B)
17 —
H3

Figure S3. Structural characterization of ASF1-SPT2 co-chaperone complex – Related to Figure 3C-E

(A) 'Global' AlphaFold prediction of the SPT2–H3.1–H4–ASF1A histone co-chaperone complex colored by PAE domains, in magenta is the region of SPT2–H3.1–H4–SPT2 related to Figure 3C-D.

(B) 'Global' AlphaFold prediction of the SPT2–H3.1–H4–ASF1A histone co-chaperone complex colored by the per residue confidence score (pLLDT) showing the accuracy of locally predicted structural elements in the model.

(C) 'Local' AlphaFold prediction of SPT2 and ASF1A histone binding domains bound to H3.1–H4 extracted from full-length alpha fold prediction, colored by Left: protein chain (SPT2: yellow; ASF1A: magenta; H3.1: red; H4; blue); Top right: PAE domain; Bottom right: pLLDT score. Related to Figure 3C-D.

(D) Alignment of local AlphaFold prediction of SPT2–H3.1–H4–ASF1A (colored as per panel C, with ASF1A omitted for clarity) to the crystal structure of SPT2–(H3.2–H4)2 (white; PDB: 5BS7). Related to Figure 3E.

(E) Alignment of 'local' AlphaFold prediction of SPT2–H3.1–H4–ASF1A (colored as per panel C) to the crystal structure of Asf1–H3–H4 (white; PDB: 2HUE) demonstrating steric clashes between ASF1A (white) and the SPT2 aC2 helix that are released by a relocalisation of the SPT2 aC2 helix in the AlphaFold prediction (yellow). Related to Figure 3E.

(F) Top: AlphaFold prediction (as shown in panel C, with ASF1-H3.1–H4: white; SPT2: yellow, and residues mutated: red). Bottom: Pull-downs of FLAG-Myc-tagged SPT2 WT or mutants (M1 and M2) compared with control purification (-) from soluble cells extracts expressing Strep-HA-ASF1b WT probed by Western blot. Representative of n = 2 biological replicates.

Figure S4

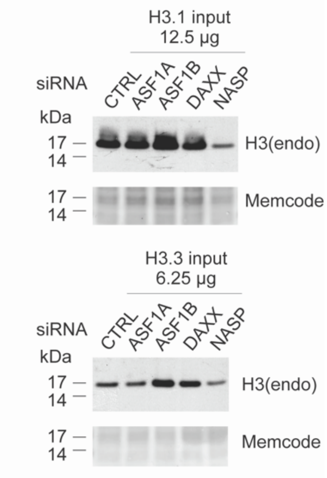

Figure S4. Effect of chaperone depletion on endogenous histone H3 levels – Related to Figure 4

Western blot of endogenous H3 in soluble extracts from cells expressing FLAG-HA tagged H3.1 (top) and H3.3 (bottom) siRNA depleted for ASF1A, ASF1B, DAXX or NASP and compared to control knockdowns siCTRL. Representative of n=2 biological replicates.

Figure S5

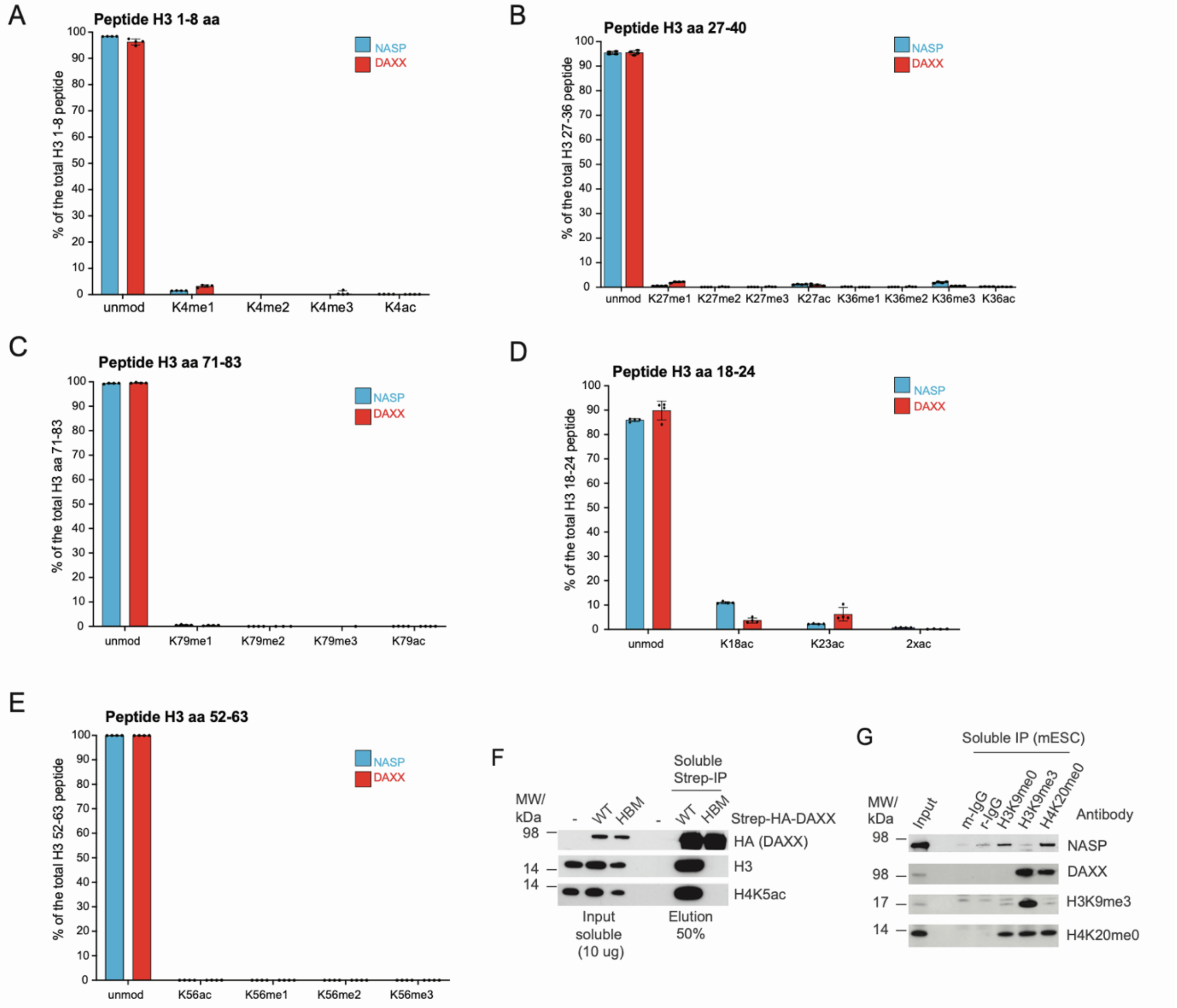

Figure S5. Additional profiling of marks on histones H3–H4 in DAXX and sNASP complexes – Related to Figure 5.

(A-E) Analysis by quantitative mass spectrometry of histone modifications as in Figure 6. The graphs show averages of four biological replicates with error bars indicating SD. PTMs were normalized using heavy peptides standards. See also Table S2.

(A) Quantification of modifications on H3 peptides 1-8.

(B) Quantification of modifications on H3 peptides 27-40.

(C) Quantification of modifications on H3 peptides 71-83.

(D) Quantification of modifications on H3 peptides 18-24.

(E) Quantification of modifications on H3 peptides 52-63.

(F) Pull-downs of Strep-HA-tagged DAXX WT or HBM compared with control purifications (−) from soluble cell extracts probed by Western blot. Representative of n=2 biological replicates.

(G) H3K9me0, H3K9me3 and H4K20me0 antibody pulldowns of endogenous histones from soluble mESC extracts compared to an IgG control, probed by Western blot. Representative of n=2 biological replicate.

Figure S6

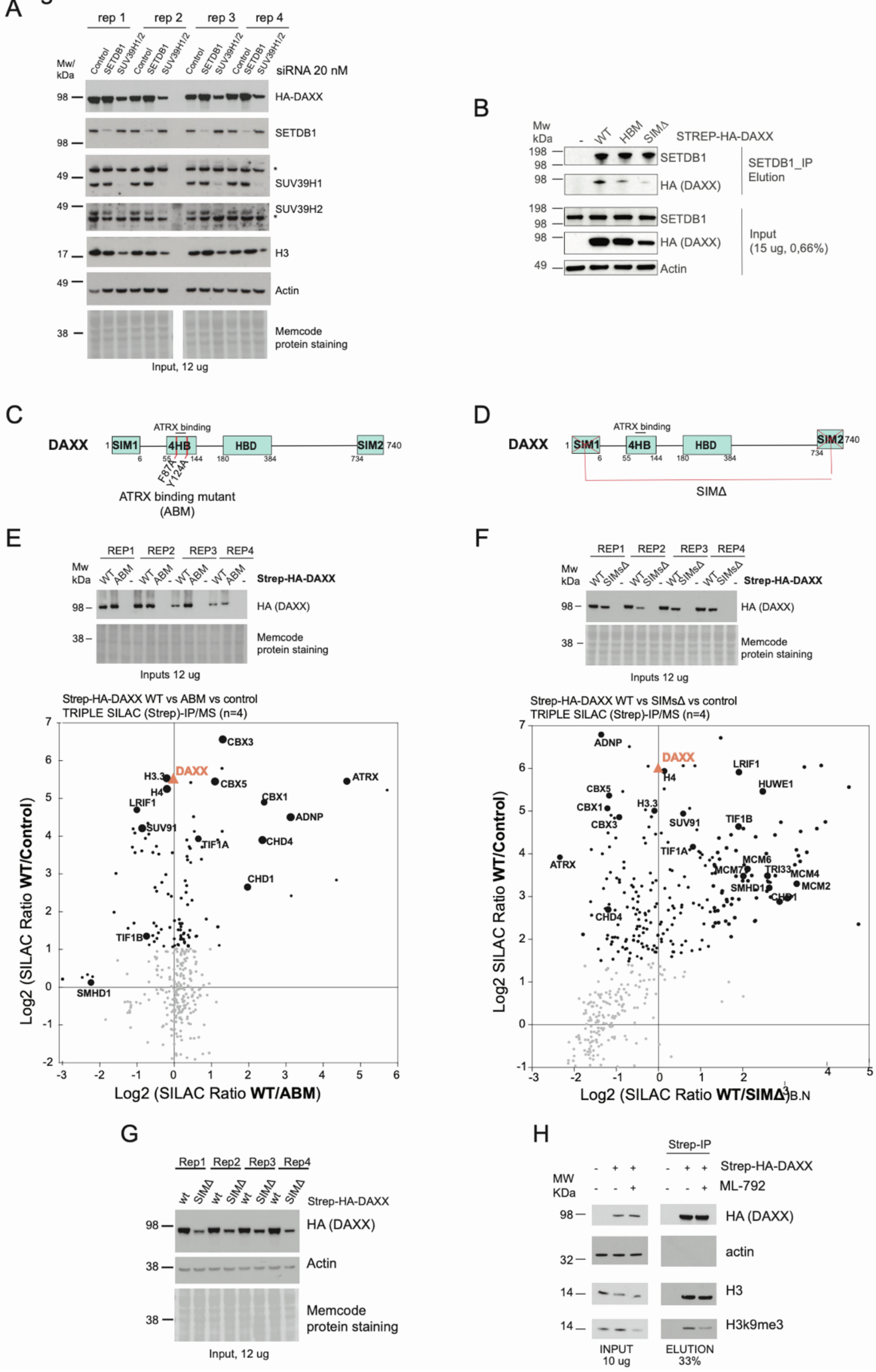
A
rep 1
rep 2
rep 3
rep 4
siRNA 20 nM
HA-DAXX
SETDB1
SUV39H1
SUV39H2
H3
Actin
Memcode protein staining
Input, 12 ug
B
STREP-HA-DAXX
SETDB1_IP Elution
Input (15 ug, 0,66%)
C
DAXX
ATRX binding
SIM1
4HB
HBD
SIM2
ATRX binding mutant (ABM)
D
SIMΔ
E
Strep-HA-DAXX WT vs ABM vs control
TRIPLE SILAC (Strep)-IP/MS (n=4)
Log2 (SILAC Ratio WT/Control)
Log2 (SILAC Ratio WT/ABM)
F
Strep-HA-DAXX WT vs SIMsΔ vs control
TRIPLE SILAC (Strep)-IP/MS (n=4)
Log2 (SILAC Ratio WT/SIMΔ)
G
Strep-HA-DAXX
HA (DAXX)
Actin
Memcode protein staining
Input, 12 ug
H
Strep-IP
Strep-HA-DAXX
ML-792
HA (DAXX)
actin
H3
H3k9me3
INPUT 10 ug
ELUTION 33%

Figure S6. Inputs and scatter plot of DAXX ABM and SIMsΔ triple SILAC IP-MS and histone PTMs experiments – Related to Figure 6.

(A) Western blot analysis of soluble extracts from cells expressing Strep-HA-tagged DAXX WT and siRNA depleted for either SEDTB1 or SUV39H1/2 and compared with Control siRNA, used to perform the DAXX associated histone PTM profiling shown in **Figure 6A**. The figure is representative of n=4 biological replicates, however, the control sample from the second replicate was removed from the MS analysis due to insufficient material. *, unspecific band.

(B) Antibody pulldowns of endogenous SETDB1 compared with control purifications (−) from soluble cell extracts expressing Strep-HA-DAXX WT, HBM or SIMΔ mutants probed by Western blot. Representative of n=2 biological replicates.

(C) Schematic domain architecture of DAXX ATRX binding mutant (ABM). Red lines indicate the point mutations in the 4-helix bundle (4HB) used to disrupt DAXX binding to ATRX.

(D) Schematic domain architecture of DAXX SIMΔ mutant showing the location of the deleted SUMO interacting motifs 1/2 (SIM1/2).

(E) (Top) Western blot analysis of soluble extracts from cells expressing STREP-HA-tagged DAXX (WT or ABM) and control cells (-), used in SILAC IP/MS pulldowns. (Bottom) Mass spectrometry analysis of SILAC labelled pull-downs of DAXX, WT and ABM and control.

(F) (Top) Western blot analysis of soluble extracts from cells expressing STREP-HA-tagged DAXX (WT or SIMΔ) and control cells (-), used in triple SILAC IP/MS pulldowns. (Bottom) Mass spectrometry analysis of SILAC labelled pull-downs of DAXX, WT and SIMΔ and control, LFQ intensities were bait normalized due to the lower expression level of DAXX SIMΔ.

(E-F) Proteins referred to by human UniProt protein identification code. Data from n=4 biological replicates. Black indicates significant enrichment over control – see statistical thresholds in **Table S1.** Related to **Figure 6E**

(G) Western blot analysis of soluble extracts from cells expressing Strep-HA-tagged DAXX WT or SIMΔ, used to perform the histone PTMs profiling. Data from n=4 biological replicates. Related to **Figure 6F**

(H) Pulldown of Strep-HA-tagged DAXX from soluble HeLa S3 extracts induced to express either DAXX WT (+) or uninduced control cells (-). Cells were co-treated with ML-792 for 6 hrs as indicated. Representative of n=2 biological replicates.

Figure S7

A

B

C

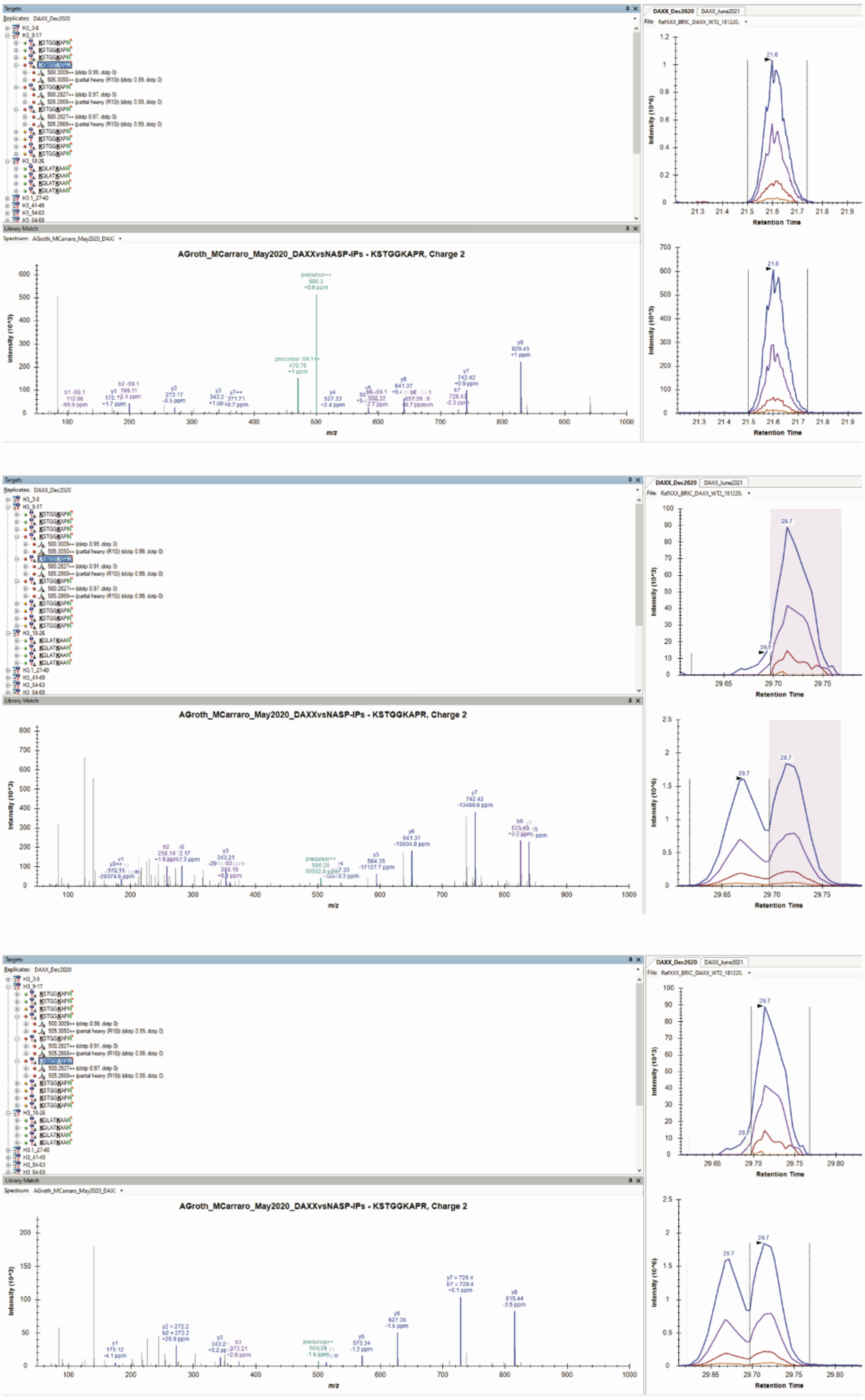

Figure S7. MS2 spectra and MS1 chromatogram of H3K9-K14 modifications – Related to Figure 5 and Figure 6. (A) MS2 spectra and MS1 chromatogram of H3K9me3. Top left panel depicts the selected modified peptide in charge state +2 (light, endogenous peptide mass: 500.30 m/z; and heavy, standard mass: 505.30) with the first lysine (K9) being tri-methylated and the second lysine (K14) unmodified (actually, artificially propionylated during sample preparation). Bottom left panel depicts the corresponding MS2, fragment spectra which proves the correct identification of the K9me3+K14unmodified peptide with low mass errors (<2.0 ppm). Right panel depicts the corresponding MS1 peaks (bounded by the dashed lines) of the modified peptide in light version (top) and heavy version (bottom), eluting around 21.6 minutes.

(B) MS2 spectra and MS1 chromatogram of H3K9ac. Top left panel depicts the selected modified peptide in charge state +2 (light, endogenous peptide mass: 500.28 m/z; and heavy, standard mass: 505.28) with the first lysine (K9) being acetylated and the second lysine (K14) unmodified (actually, artificially propionylated during sample preparation). Bottom left panel depicts the corresponding MS2, which would need to accept extremely high mass errors to be assignable (>100 ppm), thus proving that K9ac+K14unmodified peptide is absent. Right panel depicts the corresponding MS1 peaks (bounded by the dashed lines) of the modified peptide in light version (top) and heavy version (bottom), eluting around 29.7 minutes. Note the clear presence of the heavy MS1 peak given the fact that it was spiked in the samples. Note as well that this peptide is isobaric with H3K14ac.

(C) MS2 spectra and MS1 chromatogram of H3K14ac. Top left panel depicts the selected modified peptide in charge state +2 (light, endogenous peptide mass: 500.28 m/z; and heavy, standard mass: 505.28) with the first lysine (K9) unmodified (actually, artificially propionylated during sample preparation) and the second lysine (K14) being acetylated. Bottom left panel depicts the corresponding MS2, fragment spectra which proves the presence of K9unmodified+K14ace peptide given the low mass errors (<2.0 ppm). Right panel depicts the corresponding MS1 peaks (bounded by the dashed lines) of the modified peptide in light version (top) and heavy version (bottom), eluting around 29.7 minutes. Note the clear presence of both the light and heavy MS1 signals. Note as well that this peptide is isobaric with H3K9ac.

Supplemental Materials and Excel table title and legends

Supplementary Table S1. Statistically processed mass spectrometry data set, related to Figures 1B,1C, 2A-B, 3A, 4A-C, 5G, 6C, S1B-F, S2A-B, S6E, S6F.

Supplementary Table S2. Processed histone PTMs identified by mass spectrometry, related to Figures 5B-E, 6A-E, S5C-G.

Supplementary Material S1. Interactive and expandable cytoscape sessions with the histone-dependent and -independent chaperone network, related to Figure 1B and 2A.
